# Reviewing the Effectiveness of Music Interventions in Treating Depression

**DOI:** 10.3389/fpsyg.2017.01109

**Published:** 2017-07-07

**Authors:** Daniel Leubner, Thilo Hinterberger

**Affiliations:** Department of Psychosomatic Medicine, Research Section of Applied Consciousness Sciences, University Clinic RegensburgRegensburg, Germany

**Keywords:** depression, music therapy, meta-analysis, neuropsychology, psychosomatic medicine, neurophysiology, anxiety, stress

## Abstract

Depression is a very common mood disorder, resulting in a loss of social function, reduced quality of life and increased mortality. Music interventions have been shown to be a potential alternative for depression therapy but the number of up-to-date research literature is quite limited. We present a review of original research trials which utilize music or music therapy as intervention to treat participants with depressive symptoms. Our goal was to differentiate the impact of certain therapeutic uses of music used in the various experiments. Randomized controlled study designs were preferred but also longitudinal studies were chosen to be included. 28 studies with a total number of 1,810 participants met our inclusion criteria and were finally selected. We distinguished between passive listening to music (record from a CD or live music) (79%), and active singing, playing, or improvising with instruments (46%). Within certain boundaries of variance an analysis of similar studies was attempted. Critical parameters were for example length of trial, number of sessions, participants' age, kind of music, active or passive participation and single- or group setting. In 26 studies, a statistically significant reduction in depression levels was found over time in the experimental (music intervention) group compared to a control (*n* = 25) or comparison group (*n* = 2). In particular, elderly participants showed impressive improvements when they listened to music or participated in music therapy projects. Researchers used group settings more often than individual sessions and our results indicated a slightly better outcome for those cases. Additional questionnaires about participants confidence, self-esteem or motivation, confirmed further improvements after music treatment. Consequently, the present review offers an extensive set of comparable data, observations about the range of treatment options these papers addressed, and thus might represent a valuable aid for future projects for the use of music-based interventions to improve symptoms of depression.

## Introduction

“If I were not a physicist, I would probably be a musician. I often think in music. I live my daydreams in music. I see my life in terms of music.”

−Einstein, 1929.

Depression is one of the most serious and frequent mental disorders worldwide. International studies predict that approximately 322 million (WHO, 2017) of the world's population suffer from a clinical depression. This disorder can occur from infancy to old age, with women being affected more often than men (WHO, 2017). Thus, depression is one of the most common chronic diseases. Depressive suffering is associated with psychological, physical, emotional, and social impairments. This can influence the whole human being in a fundamental way. Without clinical treatment, it has the tendency to recur or to take a chronic course that can lead to loneliness (Alpass and Neville, 2003) and an increasing social isolation (Teo, 2012). Depression can have many causes that range from genetic, over psychological factors (negative self-concept, pessimism, anxiety and compulsive states, etc.) to psychological trauma. In addition, substance abuse (Neighbors et al., 1992) or chronic diseases (Moussavi et al., 2007) can also trigger depression. The colloquial use of the term “depressed” has nothing to do with the depression in the clinical sense. The ICD-10 (WHO, 1992) and the DSM-V (APA, 2013) provide a classification based on symptoms, considering the patient's history and its severity, duration, course and frequency. Within the last two decades, research on the use of music medicine or music therapy to treat depression, showed a growing popularity and several publications have appeared that documented this movement (e.g., Lee, 2000; Loewy, 2004; Esfandiari and Mansouri, 2014; Verrusio et al., 2014; Chen et al., 2016; Fancourt et al., 2016). However, most researchers used a very specific experimental setup (Hillecke et al., 2005) and thus, for example, focused only on one music genre (i.e., classical, modern; instrumental, vocal), used a predefined experimental setup (group or individual) (e.g., Kim et al., 2006; Chen et al., 2016), or specified precisely the age range (i.e., adolescents, elderly) of participants (e.g., Koelsch et al., 2010; Verrusio et al., 2014). A recent meta-analysis (Hole et al., 2015) reviewed 72 randomized controlled trials and concluded that music was a notable aid for reducing postoperative symptoms of anxiety and pain.

Dementia patients showed significant cognitive and emotional benefits when they sang, or listened to familiar songs (Särkämö et al., 2008, 2014). Beneficial effects were also described for CNMP (Chronic Non-Malignant Pain) patients with depression (Siedliecki and Good, 2006)^1^. Cardiology is an area where music interventions are commonly used for intervention purposes. Various explanations were postulated and the broad range of effects on the cardiovascular system was investigated (Trappe, 2010; Hanser, 2014). Music as a therapeutic approach was evaluated (Gold et al., 2004), and found to have positive effects before heart surgery (Twiss et al., 2006), used to increase relaxation during angiography (Bally et al., 2003), or decrease anxiety (Doğan and Senturan, 2012; Yinger and Gooding, 2015). A systematic review (Jespersen et al., 2015) concluded that music improved subjective sleep quality in adults with insomnia, verbal memory in children (Chan et al., 1998; Ho et al., 2003), and episodic long-term memory (Eschrich et al., 2008). Music conveyed a certain mood or atmosphere (Husain et al., 2002), allowed composers to trigger emotions (Bodner et al., 2007; Droit-Volet et al., 2013), based on the cultural background (Balkwill and Thompson, 1999), or ethnic group (Werner et al., 2009) someone belonged to. In contrast, the emotional state itself plays a role (Al'tman et al., 2004) on how music is interpreted (Al'tman et al., 2000), and durations are evaluated (Schäfer et al., 2013). Subjective impressions embedded in a composition caused physiological body reactions (Grewe et al., 2007; Jäncke, 2008) and even strengthened the immune system (McCraty et al., 1996; Bittman et al., 2001). The pace of (background) music (Oakes, 2003), has also been used as an essential element of many marketing concepts (North and Hargreaves, 1999), to create a relaxed atmosphere. An in-depth, detailed illustration described the wide variety of conscious, as well as subconscious influences music can have (Panksepp and Bernatzky, 2002), and endorsed future research on this subject.

### Distinction between the terms “Music Therapy [MT]” and “Music Medicine [MM]”

Most of us know what kind of music or song “can cheer us up.” To treat someone else is something completely different though. Therefore, evidence-based procedures were created for a more pragmatic approach. It is important to differentiate between music therapy and the therapeutic use of music. Music used for patient treatment can be divided into two major categories, namely [MT] and [MM], although the distinction is not always that clear.

#### Music therapy [MT]

Term used primarily for a setting, where sessions are provided by a board-certified music therapist. Music therapy [MT] (Maratos et al., 2008; Bradt et al., 2015) stands for the “…*clinical and evidence-based use of music interventions to accomplish individualized goals within a therapeutic relationship by a credentialed professional who has completed an approved music therapy program*” (AMTA)^2^. Many different fields of practice, mostly in the health care system, show an increasing amount of interest in [MT]. Mandatory is a systematic constructed therapy process that was created by a board-certified music therapist and requires an individual-specific music selection that is developed uniquely for and together with the patient in one or more sessions. Therapy settings are not limited to listening, but may also include playing, composing, or interacting with music. Presentations can be pre-recorded or live. In other cases (basic) instruments are built together. The process to create these tailor-made selections requires specific knowledge on how to select, then construct and combine the most suitable stimuli or hardware. It must also be noted that music therapy is offered as a profession-qualifying course of study.

#### Music medicine [MM] (i.e., functional music, music in medicine)

Carried out independently by professionals, who are not qualified music therapists, like relaxation therapists, physicians or (natural) scientists. A previous consultation, or collaboration, with a certified music therapist can be helpful (Register, 2002). In recent years, significant progress has been made in both the research and clinical application of music as a form of treatment. It has valuable therapeutic properties, suitable for the treatment of several diseases. The term “music medicine” is used as a term for the therapeutic use of music in medicine (Bradt et al., 2015, 2016), to be able to differentiate it from “music therapy.” [MM] stands for a medical, physiological and physical evaluation of the use of music. If someone listens to his or her favorite music, this is sometimes also considered as a form of music medicine. [MM] deliberately differs from music therapy as part of psychiatric care or psychotherapy. It is important to stress out that the term “Music Therapy [MT]” should not be used for any kind of treatment involving music, although there is without doubt a relationship between [MT] and [MM]. What all of them have in common is the focus on a scientifically, artistically or clinically based approach to music.

#### “Seamless Transitions” between music therapy [MT] and music medicine [MM]

Activity used for treatment is ambiguous or not clearly labeled as “Music Therapy” or “Music Medicine.” It should not be forgotten that the definition of “Music Therapy” is not always clearly distinguishable from “Music Medicine.” One possible scenario would be a physician (i.e., “non-professional”), who is not officially certified by the AMTA (or comparable institutions), but still acts according to the mandatory rules. In addition, depending on one's home country, uniform standards or eligibility requirements might be substantially different. We think that every effort should be recognized and therefore postulate one definition that can describe the main principle of [MT], [MM], and everything in between, in one sentence: “*Implementation of acoustic stimuli (“music”) as a medium for the purpose of improving symptoms in a defined group of participants (patients) suffering from depression.”*

## Materials and methods

### Literature search

Search strategy and selection process was performed according to the recommended guidelines of the Cochrane Centre on systematic literature search (Higgins and Green, 2008). Our approach (**Figure 2**) was according to their scientific relevance, supplemented by the analysis of relevant journals, conferences and workshops of recent years. We obtained 60,795 articles from various search engines as initial result. Retrieved data was collected and processed on an existing personal computer with the latest Windows operating system.

#### Search, collection, selection, and review strategies

We used a combination of words defining three search-categories (Music-, Treatment-, and Depression associated) as well as several words (e.g., Sound, Unhappy, and Treatment) assigned to each category as described in the collection process.below. If synonyms of those keywords were identified, they were added as well. Theme-categories^3^ were created next, then related keywords identified and added into a table. “Boolean Operators^4^” were used as logical connectives to broaden and/or narrow our search results within many databases (mostly search engines as described below).

This way the systematic variation of keyword-based queries and search terms could be performed with much more efficiency. To find the most relevant literature on the subject, keywords were entered into various scientific search engines, namely PubMed, MEDLINE, and Google Scholar. After the collection process, several different steps were used to reduce the number of retrieved results. Selection out of the collected material included to narrow down search results to a limited period of time. We decided to choose a period between 1990 and 2016 (i.e., not exceeding 26 years), because within these years several very interesting works of research were published, but often not mentioned explicitly, discussed in detail, or the main target of a comparative review. After several papers were excluded, a systematic key phrases search was conducted once more to retrieve results, limited to original research articles^5^. We also removed search results that quoted book chapters, as well as reports from international congresses and conferences. Research papers that remained were distinguished from duplicates (or miss-matches not dismissed yet). Based on our predefined criteria for in- and ex-clusion, relevant publications were then selected for an intensified review process. Our plan was to apply the following inclusion criteria: Original research article, published at time of selection, music and/or instruments were used intentionally to improve the emotional status of participants (i.e., intended or officially confirmed as music therapy). The following exclusion criteria were used: No original research, article was not published (e.g., project phase, in review), unverified data or literature was used, participants did neither receive nor interact with music. Not relevant for in- or exclusion was the kind of questionnaire used to measure depression, additional diagnostic measures for pathologies other than depression, spatial and temporal implementation of treatment, demographics (i.e., number, age, and gender) participants had, or distinctive features (like setting, duration, speakers, live version, and recorded) of stimuli. After the initial number of results, the remaining articles were manually checked for completeness and accuracy of information. Our final selection of articles included 28 research papers.

### General information — (Figure 1)

#### Evaluating the methodological quality of our meta-review

During the review process, we used a very strict self-monitoring procedure to ensure that the quality of scientific research was met to the best of our knowledge and stood in accordance with the standards of good scientific practice. Every effort has been made to provide the accuracy of contents as well as completeness of data published within our meta-review. Inspired by another author's meta-review (Kamioka et al., 2014), we evaluated our work by the AMSTAR checklist (Shea et al., 2007)^6^ and found no reasons for objection regarding our selection of reviews. AMSTAR (acronym for “Assessment of Multiple SysTemAtic Reviews”), a questionnaire for assessing systemic reviews, is based on a rating scale with 11 items (i.e., questions). AMSTAR allows authors to determine and graduate the methodological quality of their systematic review.

**Figure 1:**
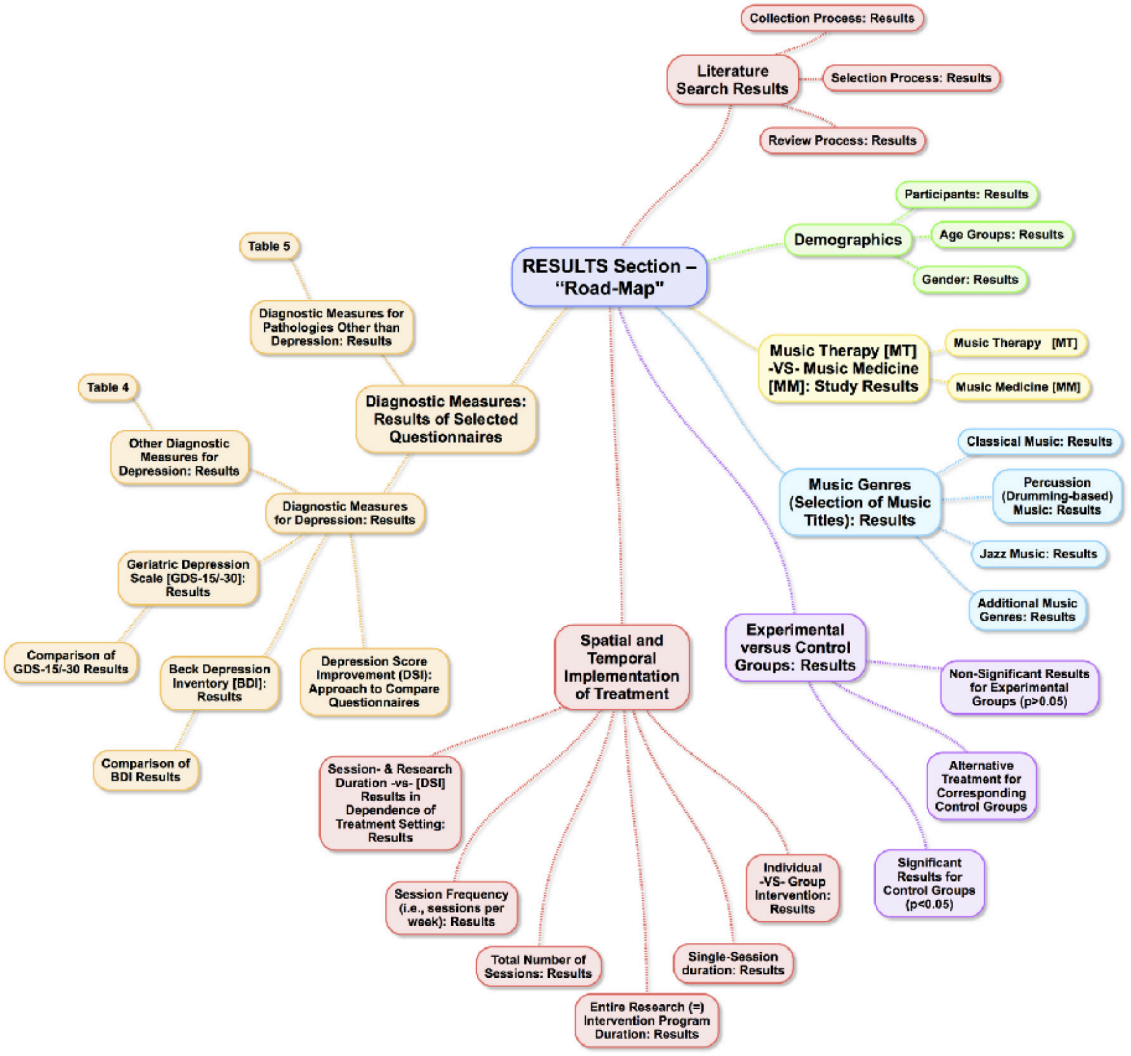
“Road-Map” Outline of the following results section (idea, concept and creation of this Figure by Leubner).

#### Effect size

We investigated a wide variety of scholarly papers within our review. There were many different approaches and several procedures. As far as intervention approaches and procedures were concerned, we found (very) similar trends in several papers. To ensure that those different tendencies were not only based on our pure assumption as well as biased interpretation, we also calculated the effect-size correlation by using the mean scores as well as standard deviations for each of the treatment and control groups, if this setup was used by the respective researcher. Most trials showed a small difference in between the experimental and control group at baseline, what almost always turned into a large effect size regarding post-measurement.

#### Depression score improvement (DSI) — approach to compare questionnaires

As mentioned above, we selected 28 scholarly articles that used different questionnaires to measure symptoms of depression for experimental and control groups. According to common statistical standards we used a formula to evaluate and compare the relative standing of each mean to every other mean. To avoid confusion, we decided to refer to it as “Depression Score Improvement (DSI).” Mathematically speaking it stands for the mean difference between the pre-test and post-test results (i.e., score changes) in percent. (DSI_Ind_) stands for an individual and (DSI{_Gr_) for a group setting. Please refer to the Supplementary Materials (Table: “Complete Display of Statistical Data”)^7^ for additional information.

## Results

The results will review the works in terms of demographics, treatment implementation, and diagnostic measures.

### Literature search results — (Figure 2)

#### Collection process – results

A large list of keywords, based on several questions we had, was created initially. They were combined into search-terms and finally put into search-categories as category-dependent keywords. In addition, we discussed several parameters and agreed on three categories (associated to music, treatment, and depression). By querying scientific databases, using the above-mentioned category-dependent keywords as input criteria, we retrieved a very large number of results. We then searched for a combination of the following words and/or phrases (e.g., “*music AND therapy AND depression”; “acoustic AND intervention AND unhappy”*), narrowed down the retrieved results according to a combination of several keywords (e.g., “*music therapy”; “acoustic intervention”*), and sorted this data according to relevance.

**Figure 2:**
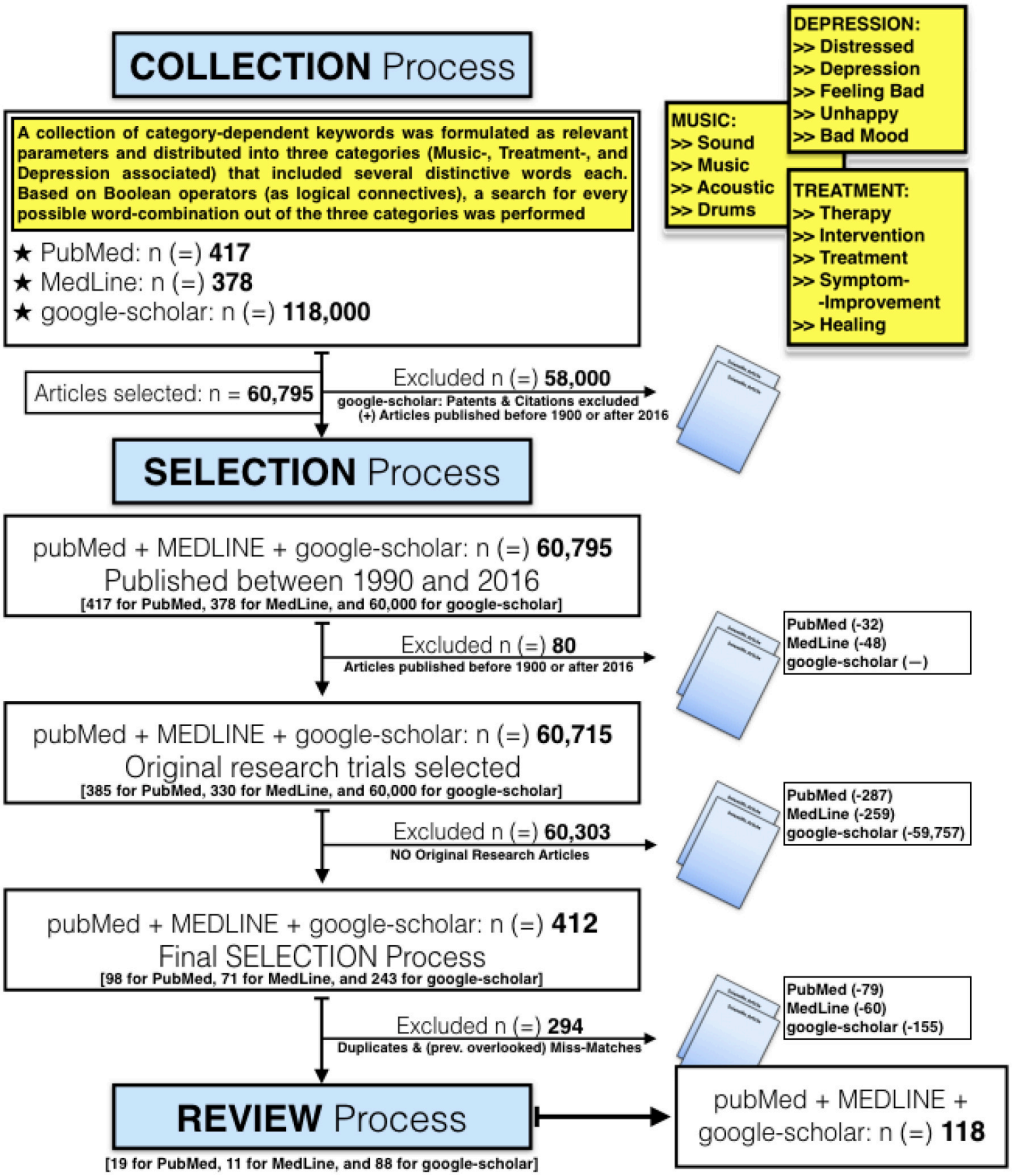
Overview of our Collection, Selection, and Review Process (idea, concept and creation of this Figure by Leubner). Initially, the total number of retrieved results was 118,000 as far as google-scholar was concerned. Analysis was complicated by the disproportionately high number of results from google-scholar. Therefore, we decided to narrow down this initial search query to a period from 1990 up to 2016, and reduced the results from google-scholar to 60,000 this way. Compared to the other two search engines, this process was done two steps ahead. At google-scholar we excluded patents as well as citations in the initial window for our search results. Unfortunately search options are very limited, and though we retrieved at first this overwhelming number of 118,000 results!. Some keywords (e.g., anxiety, pain, fear, violence) were deliberately excluded right from the beginning. This was done right at the start of our selection/search process, to prevent a systematic distortion of retrieved results.

#### Selection process – results

In step two we applied the above-mentioned approach and narrowed down our search query to a limited period of time, then systematically searched for key phrases, and excluded duplicates as well as previously overlooked miss-matches. Our inclusion criteria can be summarized as follows: Original research article, already published at time of selection, music and/or instruments were used intentionally to improve the emotional status of participants. Our exclusion criteria were: No original research, article was not published (e.g., project phase, in review), unverified data or literature was used, participants did neither receive nor interact with music.

#### Review process – results

Based on our predefined criteria for inclusion and exclusion, relevant publications were then selected and used for our intensified review process. After reducing the initial number of results, we obtained the remaining articles, conducted a hand-search in selected scientific journals, and manually checked for completeness as well as accuracy of the contained information. The final selection of articles, according to our selection criteria, included 28 papers.

### Demographics^8^

To begin with, the number of participants as well as age and gender related basic demographics were analyzed.

#### Participants – results

Our final selection of 28 studies included 1,810 participants, with group sizes between five and 236 persons (n_av_ = 64.64; *SD* = 56.13). For experimental groups, we counted 954 individuals (*n*_min_ = 5; *n*_max_ = 116; *n*_av_ = 34.07; *SD* = 27.78), and 856 (*n*_min_ = 10; *n*_max_ = 120; *n*_av_ = 30.57; *SD* = 29.10) for the control respectively. Although three authors (Ashida, 2000; Guétin et al., 2009b; Schwantes and Mckinney, 2010) did not use a control sample, those articles were nevertheless considered for calculating accurate and up-to-date data. Depending on each review, sample groups differed profoundly in number of participants. The smallest one had five participants (Schwantes and Mckinney, 2010), followed by three authors (Hendricks et al., 1999; Ashida, 2000; Guétin et al., 2009b) who used between 10 and 20 individuals in their clinical trials. Medium sized groups of up to 100 participants were found in six articles (Gupta and Gupta, 2005; Castillo-Pérez et al., 2010; Erkkilä et al., 2011; Wang et al., 2011; Lu et al., 2013). Large groups with more than 100 (Koelsch et al., 2010; Silverman, 2011), or 200 (Chen et al., 2016) participants were the exception, and 236 participants (Chang et al., 2008) presented the upper end in our selection.

#### Age groups – results

Within our selected articles, the youngest participant was 14 (Hendricks et al., 1999), and the oldest 95 years of age (Guétin et al., 2009a). We then separated relevant groups, according to their age, into three categories, namely “young,” “medium,” and “elderly.”

##### Young

Participants were defined as “young,” if their mean age was below or equal to 30 years (≤30). Young individuals did show minimal better (i.e., higher) depression score improvements (DSI) (mean difference between the pre-test and post-test results was calculated in percent), if they attended group (mean DSI_Gr_ = 53.83%)^9^, rather than individual (DSI_Ind_ = 40.47%) music intervention sessions. These results may be due to the beneficial consequences of social interactions within groups, and thus confirm previous study results (Garber et al., 2009; Tartakovsky, 2015).

##### Medium

We used the term “medium” for groups of participants, whose mean ages ranged between 31 (>30) and 59 years (<60). Medium-aged participants presented much better results (i.e., higher depression score improvements), if they attended a group (mean DSI_Gr_ = 48.37%), rather than an individual (mean DSI_Ind_ = 24.79%) intervention setting. However, it should be stressed that our findings only show a positive trend and thus should not be evidence.

##### Elderly

The third and final group was defined by us as “elderly” and included participants with a mean age of 60 years or above (≥60). Noticeable results were found for the age group we defined as elderly, as participants showed slightly better (i.e., higher) score improvements (mean DSI_Ind_ = 48.96%), if they attended an individual setting. Considering the music selection that had been used for elderly participants, a strong tendency toward classical compositions was found (e.g., Chan et al., 2010; Han et al., 2011). Because a relevant number of participants came from Asian countries (e.g., China, Korea), elderly people from those research articles received, in addition to classical music, quite often Asian oriented compositions as well. Despite our extensive investigations, the influence this combination had on results, remained uncertain. Positive tendencies within those groups might be due to “traditional” and/or “culture related” factors. It is, however, also conceivable that combining Western classical with traditional Asian music is notably suited to produce better results. Concerning this matter, future research on western depression patients treated with a combination of classical Western, and traditional Asian music might be a promising concept to be further explored.

#### Gender – results

As far as gender was concerned, we subdivided each sample group in its female and male participants. Women and men were found in 20 study designs. This was the most frequently used constellation. Within this selection, we did not find any significant differences, and so no further analysis was done. Only women took part in two studies (Chang et al., 2008; Esfandiari and Mansouri, 2014)^10^. Interestingly the same stimuli setup was used in both cases. It consisted of instrumental music without vocals, stored on a digital record, and was presented via loudspeakers from a CD (Chang et al., 2008) or MP3 player (Esfandiari and Mansouri, 2014). Only men were seen in four research papers (Gupta and Gupta, 2005; Schwantes and Mckinney, 2010; Albornoz, 2011; Chen et al., 2016). A significant improvement of depression scores was reported for every experimental group, and once (Albornoz, 2011) for a corresponding control setting (received only standard and no alternative treatment). Three articles (Schwantes and Mckinney, 2010; Albornoz, 2011; Chen et al., 2016) shared several similarities, as percussion instruments (e.g., drums, tambourines) were part of each genre selection, all participants received music interventions in a group setting, and stimuli were actively produced within a live performance. In addition, the BDI questionnaire has also been used in three cases (Gupta and Gupta, 2005; Albornoz, 2011; Chen et al., 2016), and thus we were able to perform a search for similarities or tendencies. The average duration for one music intervention was 80 (*SD* = 45) min and the total number of sessions was 17 (*SD* = 5) in average. Two publications (Hsu and Lai, 2004; Wang et al., 2011) did not offer any information about gender related distribution of participants.

### Music therapy [MT] vs. music medicine [MM] — study results

#### Music-therapy [MT]

Within our selection of 28 articles, six explicitly mentioned a certified music therapist (Hanser and Thompson, 1994; Choi et al., 2008; Schwantes and Mckinney, 2010; Erkkilä et al., 2011; Han et al., 2011; Silverman, 2011)^11^. For five articles with available data, a combined average depression score improvement (DSI) of 40.87% (*SD* = 7.70%) was calculated for the experimental groups. As far as the relevant control groups were concerned, only twice depression scores decreased at all (Choi et al., 2008; Erkkilä et al., 2011; Table 1).

**Table 1:** Music-Therapy interventions—music types and results.

**Author and year**	**Intervention**		**Depression measurement methods**								
	**Music type**	**[MT] or [MM]**	**Test**	**Experimental [mean *(SD)*]**				**Control [mean (*SD*)]**			
				**Pre *(SD)***	**Post *(SD)***	**Change (%)**	***p*-Level**	**PRE *(SD)***	**POST *(SD)***	**Change [%]**	***p*-Level**
Choi et al., 2008	Drumming, Relax-Music, Singing	[MT]	BDI	49.30 (3.10)	25.50 (2.20)	48.28	*p* < 0.001	47.40 (2.80)	44.80 (3.80)	5.49	*p* > 0.05
Erkkilä et al., 2011	Drumming (Djembe Drum)	[MT]	MADRS	24.60 (6.40)	14.10 (8.77)	42.68	*p* < 0.05	23.00 (7.60)	16.43 (9.33)	28.57	*p* > 0.05
Han et al., 2011	Drumming Singing, Dancing, Improvisation with various Instruments	[MT]	RMBPC_D_	20.50 (23.5)	11.70 (15.9)	42.93	*p* < 0.05	13.10 (21.0)	24.60 (34.70)	−87.79	*p* > 0.05
			AES	18.20 (6.40)	19.0 (4.80)	−4.40	*p* > 0.05	17.10 (4.30)	16.60 (5.10)	2.92	*p* > 0.05
Hanser and Thompson, 1994	Relax-Music, Improvisational Harp Music, PMR (spoken cues)	[MT] Visit	GDS-30	17.30 (5.85)	7.70 (3.66)	55.49	*p* < 0.05	15.30 (5.85)	16.20 (6.13)	−5.88	*p* > 0.05
		[MT] Call	GDS-30	17.60 (7.89)	12.30 (8.65)	30.11	*p* < 0.05				
Schwantes and Mckinney, 2010	Drumming, Guitar, Piano	[MT]	CES-D	21.60 (3.22)	15.60 (2.66)	27.78	*p* < 0.05	–	–	–	–
Silverman, 2011	12-bar BLUES, Blues Songwriting	[MT]	BDI	n/a	18.79 (9.14)	(–?–)	*p* > 0.05	n/a	20.28 (9.53)	(–?–)	*p* > 0.05

Regarding the kind of music provided by a board-certified music therapist, we found some similarities that stood out and appeared more frequently, when compared to music medicine interventions. Percussion music (mainly drumming) was used by four researchers (Choi et al., 2008; Schwantes and Mckinney, 2010; Erkkilä et al., 2011; Han et al., 2011). One author (Choi et al., 2008) used music based on instruments that were selected according to participant's preferences. Included were, for example, egg shakes, base-, ocean-, and paddle-drums. Participants actively played and passively listened to instruments or sounds, complemented by singing together. Another researcher (Erkkilä et al., 2011) preferred the African Djembe^12^ drum as well as a selection of several percussion sounds created digitally by an external MIDI (*Musical Instrument Digital Interface*) synthesizer. Percussion-oriented improvisation that included rhythmic drumming and vocal patterns was another approach one scholar (Han et al., 2011) used for his stimuli selection. Congas, Cabassas, Ago-Gos, and Claves was the percussion-based selection (in addition a guitar and a Piano was also available) in the fourth music-therapy article (Schwantes and Mckinney, 2010). Twice, music without the use of percussion instruments or drums in general, was selected for the intervention. Once (Hanser and Thompson, 1994) relaxing, slow and rhythmic harp-samples, played from a cassette-player, were used. In addition, each of the participants was invited to bring some samples of her or his favored music titles. The second one (Silverman, 2011) decided to play a “12-bar Blues” (i.e., “blues changes”)^13^ progression as an introduction, followed by a Blues songwriting session. The last-mentioned music-therapy project was the only article out of six, where participants within their respective music intervention group did not present a significant reduction of depression. A very interesting “fund” was that none of the music-therapy articles neither concentrated their main music selection on classical, nor on Jazz music. When we looked for other distinctive features it turned out that stimuli were actively produced within a live performance in five articles. There was only one exception (Hanser and Thompson, 1994), where a passive presentation of recorded stimuli was preferred by the scholar.

#### Music-medicine [MM]

The remaining 22 research articles did not explicitly mention a certified music therapist. In those cases, some variant of music medicine was used for intervention. Often the expression music therapy was used, although a more detailed description or specific information was neither published nor available upon our request. With one exception (Castillo-Pérez et al., 2010), we could calculate the (DSI)^9^ for 25 articles that used some variant of music-medicine [MM].

When we investigated the kind of music that was used, a broader selection of genres was found. Percussion based tracks and drumming appeared in five scholarly papers (Ashida, 2000; Albornoz, 2011; Lu et al., 2013; Chen et al., 2016; Fancourt et al., 2016). Researchers that used drums reported a significant depression score improvement for every experimental group and we calculated an average of 53.71% for those five articles. Regarding the kind of genre used in our selection of music-medicine articles, a wider range of genres was found. One of the biggest differences was that only music-medicine articles used, in addition to percussion stimuli, also classical and Jazz music for their intervention. Please note that for reasons of confusion, we do not mention the Seamless Transitions between Music Therapy [MT] and Music Medicine [MM] from the “Materials and Methods Section.”

### Music genres (selection of music titles) – results

Regarding the kind of music used in our selection of research articles, a wide range of genres was found. Mainly three styles, classical^14^ (9x), percussion^15^ (9x), and Jazz (5x) music were used more frequently for music intervention. The evaluation took place when specific compositions showed significantly greater improvements in depression compared to other research attempts. Utilizing our comprehensive data analysis, music titles were categorized according to genre or style (e.g., classical music, Jazz), narrowed down (e.g., Jazz), sorted by magnitude of depression score improvements (DSI)^9^, and finally examined for distinctive features (like setting, duration, speakers, live version, recorded). Similarities that stood out and appeared more frequently among one selected music genre were compared with the 28 scholarly articles we selected for our meta-review.

#### Classical music – results

In nine articles, classical music (Classical or Baroque period)^22^ was used. Several well-known composers such as W.A. Mozart (Castillo-Pérez et al., 2010), L. v. Beethoven (Chang et al., 2008; Chan et al., 2009) and J. S. Bach (Castillo-Pérez et al., 2010; Koelsch et al., 2010) have been among the selected samples. If classical music was used as intervention, our calculations revealed that four studies out of eight^16^ were among those with depression score improvements (DSI)^31^ that were above the average^17^ of 39.98% (*SD* = 12). When we looked for similarities between these, three of the four studies (Harmat et al., 2008; Chan et al., 2009; Guétin et al., 2009a) used individual sessions, rather than a group setting (Koelsch et al., 2010). For all four articles mentioned above, we calculated an average of 11 (*SD* = 10) for the total number of sessions that included classical music. The remaining five articles on the other hand, presenting results not as good as the aforementioned, showed an average of 30 (*SD* = 21) music interventions. One plausible hypothesis might be “saturation effect” caused by too many interventions in total. Too little variety within the selection of music titles has probably played an important role as well. A general tendency that less intervention sessions in total would lead to better results for every case where classical compositions were included could not be confirmed for our selection.

#### Percussion (drumming-based) music – results

Percussion music (mainly drumming) was used by nine^18^ researchers, and among those, two ways of integration were found. On the one hand, rhythmic percussion compositions were included as part of the music title selection used for intervention. On the other hand, and this was the case in nine articles, various forms of drums had been offered to those who joined the experimental groups, allowing them to “produce their own” music. Sometimes participants were accompanied by a music therapist (e.g., Albornoz, 2011) or professional artist (Fancourt et al., 2016), who gave instructions on how to use and play these instruments. When we looked for trends or distinctive features percussion music (in particular drumming) had, it turned out that, except one article (Erkkilä et al., 2011), all were carried out within a group, rather than an individual setting. A further search for additional similarities, leading to better outcome scores, did not deliver any new findings as far as improvement of depression was concerned. Participants in altogether 7 out of 9 percussion groups were medium aged, two authors (Ashida, 2000; Han et al., 2011) described elderly participants, whereas none of the percussion groups included young participants.

A wide and even distribution of reduced depression scores across all outcome levels became apparent, when participants received percussion (or drumming) interventions. We calculated an average depression score improvement (DSI) of 47.80% (*SD* = 14). Above-average results regarding depression score improvement (DSI), were achieved in four experiments that had an average percussion session duration of 63 (*SD* = 19) min. In comparison, we calculated for the remaining five articles an average of 93 (*SD* = 26) min. Although a difference of 30 min showed a clear tendency, it was not enough of a difference to draw any definitive conclusions.

#### Jazz music – results

Finally, five^19^ researchers used primarily Jazz^20^ as music genre for their intervention. Featured performers (artists) were Vernon Duke (“April in Paris”) (Chan et al., 2009), M. Greger (“Up to Date”), and Louis Armstrong (“St. Louis Blues”) (Koelsch et al., 2010). Unfortunately, available data was quite limited, mainly since most authors did not disclose relevant information and a detailed description was rarely seen. Some interesting points were also found for research articles that used Jazz as a treatment option. All five of them were among those with good outcome scores, as far as depression reduction was concerned. Test scores ranged between a significance level of *p* < 0.01 (Guétin et al., 2009a; Verrusio et al., 2014; Chen et al., 2016) and sometimes even better than *p* < 0.001 (e.g., Koelsch et al., 2010; Fancourt et al., 2016). Depression score improvement (DSI) had an average of 43.41% (*SD* = 6). However, there was no clear trend leading toward Jazz as a more effective intervention option, when compared to other music genres. This was assumed because the two studies that showed the best^21^ reduction in depression [Chan et al., 2010 (DSI = 48.78%); Koelsch et al., 2010 (DSI = 4 6.58%)] used both classical music in addition to Jazz as an intervention. Experimental groups received two types of intervention (i.e., classical music and Jazz) which eventually blurred outcome scores or prevented more accurate results. Since it was not possible to differentiate to what extent either classical music or Jazz was responsible for the positive trend in reducing symptoms of depression, further research in this field is needed.

#### Additional music genres – results

Numerous other music styles were used in the experiments, ranging from Indian ragas^22^ played on a flute (Gupta and Gupta, 2005; Deshmukh et al., 2009), nature sound compositions (Ashida, 2000; Chang et al., 2008), meditative (Chan et al., 2010), or slow rhythm music (Chan et al., 2012), to lullabies (Chang et al., 2008), pop or rock (Kim et al., 2006; Erkkilä et al., 2011), Irish folk, Salsa, and Reggae (Koelsch et al., 2010), only to name a few. As far as we were concerned all those genres mentioned above would present interesting approaches for future research. Due to a relatively small number and simultaneously wide-ranging variety, more thorough investigations are needed, though. These should be examined independently. As far as the above-mentioned music genres, other than classical, percussion, or Jazz were concerned, no indication for a preferable combination was observed.

### Experimental vs. control groups – results

#### Non-significant results for experimental groups (*p* > 0.05)

In two (Deshmukh et al., 2009; Silverman, 2011) out of 28 studies within our selection of research papers, no significant reduction in depression scores was reported, after participants participated in music interventions. Within those two cases all relevant statistical observations differed without any obvious similarities indicating reasons for non-significant results. Although the results did not meet statistical significance for symptom improvement, both authors explicitly pointed out that positive changes in the severity of depression became obvious for the respective experimental groups. We declared one article (Guétin et al., 2009b) as significant, although it was marked as non-significant in our complete table. This was due to the overall results of this specific research paper, with significant [HADS-D] test scores for weeks 5, 10, and 15. Only week 20 did not follow this positive trend of improvement. It is also important to mention that after music treatment every one of the additional tests [HADS-A for Anxiety; Face(-Scale) to measure mood] showed significant improvements for the experimental group.

#### Alternative treatment for corresponding control groups

Control groups, who received an alternative (i.e., non-music) intervention, were found in nine research articles (e.g., Guétin et al., 2009a; Castillo-Pérez et al., 2010)^23^. We investigated whether there were particularly noticeable differences in outcome scores, when relevant control groups, who received an alternative treatment, were compared to those who received no additional intervention at all (or only the usual treatment)^24^. As far as these nine articles were concerned, a significant reduction (*p* < 0.05) in depression scores was found in every experimental but only one control setting (Hendricks et al., 1999). In this case, an entirely different result became apparent, when control participants received a Cognitive-Behavioral Therapy [CBT] and a significant reduction (*p* < 0.05) in depression scores was measured compared to the respective baseline score, although music still lead to better results. Another scholar (Chan et al., 2012)^25^, instructed participants in the control group to take a resting period, while simultaneously the experimental attendees joined their music intervention session. This alternative approach did not reduce the [GDS-15] depression score, but even increased it. Interestingly, the same author previously published (Chan et al., 2009) a significant (*p* = 0.007) increase (i.e., worsening of depression) for the relevant control setting. To be complete, a resting period was also conducted in another case (Hsu and Lai, 2004), but results showed also no significant reduction in depression scores. Other attempts to provide an alternative intervention for the control group have been monomorphic tones (Koelsch et al., 2010) that corresponded to the experimental music samples (in pitch-, BPM-, and duration), verbal treatment sessions (Silverman, 2011), antidepressant drugs (Verrusio et al., 2014)^26^, reading sessions (Guétin et al., 2009a) or a “conductive-behavioral” psychotherapy (Castillo-Pérez et al., 2010).

#### Significant results for control groups (*p* < 0.05):

Significant reduction of depression (*p* < 0.05) in corresponding control (“non-music treatment”) groups was reported twice (Hendricks et al., 1999; Albornoz, 2011) within our selection of scholarly articles. In one instance (Albornoz, 2011) the relevant participants received only standard care, but in the other case (Hendricks et al., 1999) an already above mentioned alternative treatment (i.e., “Cognitive-Behavioral Activities”) was reported.

### Spatial and temporal implementation of treatment

#### Individual vs. group intervention – results

As postulated by previous literature (Wheeler et al., 2003; Maratos et al., 2008), we differentiated mainly two scenarios based on the number of participants who attended music intervention sessions and referred to them as “group” or “individual.” Group sessions can awaken participants' social interactions and individual sessions often provides motivation (Wheeler et al., 2003). Here, a “group” scenario was specified, if two or more persons (*n* ≥ 2) were treated simultaneously, whereas “individual” determined experimental settings where only one single person received music interventions individually (*n* = 1). Among our article selection we could find a well-balanced distribution of 15 trials with participants who received music interventions in a group, while 13 researchers used an individual setting. First, the impact of individual compared to group treatment was evaluated. Here an almost equivalent outcome (for the significance-level of results) across all 13 individual, compared to 15 group settings was found, without any advantage to one over the other. Non-significant improvements were seen once for a group (Silverman, 2011) and once^27^ for an individual (Deshmukh et al., 2009) intervention.

#### Single-session duration – results

**Figure 3:**
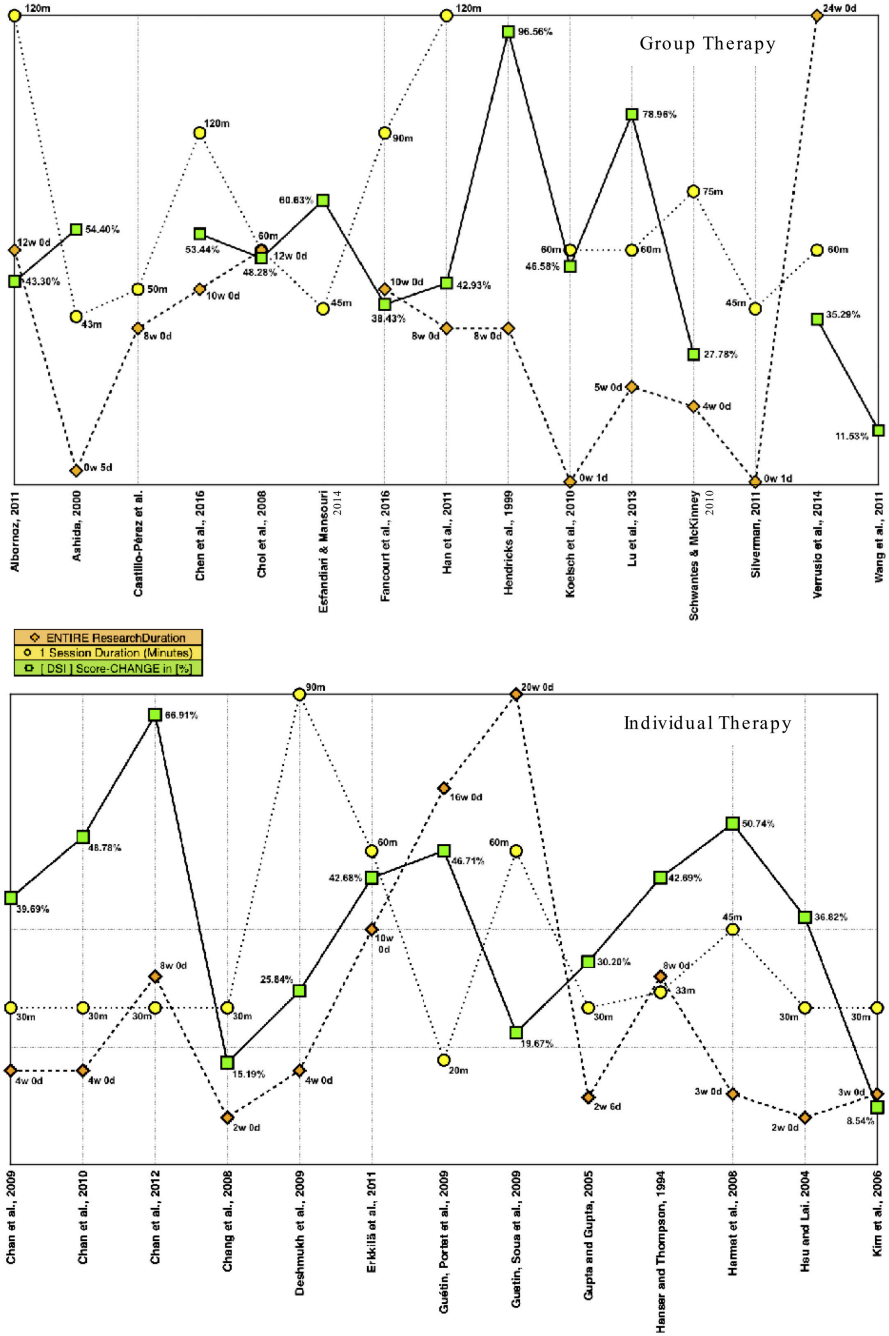
Session- and research duration–vs.–[DSI] results in dependence of treatment setting.

The question whether groups showed different (i.e., more or less) improvements, if the duration of one single session was altered, we decided to use the intervention length as a key metric (Figure 3). Except for two instances (Hendricks et al., 1999; Wang et al., 2011), 26 research papers reported the duration one single treatment had. Among those 20 min (Guétin et al., 2009a) was the shortest, and 120 min (Albornoz, 2011; Han et al., 2011) the longest duration for one session. The average for all 26 articles was 55 min, 70 min for 13^28^ group settings, and 40 min as far as the 13 individual intervention setups were concerned.

#### Entire research (=) intervention program duration – results

Continuing our review process, some interesting diversity was found for the scheduled (i.e., total) treatment duration (Figure 3). It ranged from 1 day in two cases (Koelsch et al., 2010; Silverman, 2011) up to 20 (Guétin et al., 2009b), or even 24 weeks (Verrusio et al., 2014). Out of 26 trials an average duration of 7 weeks was found. In two cases, the data was missing (Wang et al., 2011; Esfandiari and Mansouri, 2014). The scheduled (i.e., total) treatment duration was determined by a variety of factors. Our investigation, whether there was any relationship between the entire duration of experimental projects and relevant outcome scores, delivered the following results. For an individual (Ind) therapy setting, we isolated eight^29^ research papers with above average^30^ results in depression score improvement (DSI_Ind_ > 36.50%). We then calculated for the entire project an average duration of almost 7 weeks. For the remaining five^31^ articles that also used an individual approach, but had below average depression score improvements, an average duration of 6 weeks was found. A different picture became apparent when we selected those four^32^ articles that presented better than average (DSI_Gr_ > 49.09%) results in depression score improvement, after participants received music intervention in a group (Gr). Percussion music (mainly drumming) was used by three researchers (Ashida, 2000; Lu et al., 2013; Chen et al., 2016). In comparison, the fourth author (Hendricks et al., 1999) used a selection of relaxing music for treatment. For this setup, a combined duration of six (*SD* = 4) weeks was calculated for the entire project length. On the other hand, a mean close to 10 (*SD* = 7) weeks was found for the remaining 7^33^ group intervention projects that were less successful (i.e., below average), as far as depression score reduction was concerned. Based on these results, we concluded that the length for the entire music intervention procedure might be a crucial element for successful results, and seems to be associated with the intervention type. These findings were not enough to draw further conclusions for every project though, but as far as our selection was concerned, a slightly longer intervention duration of 7 weeks led to better results if participants were treated individually. In comparison, for a group setting our calculations revealed a different picture, when we calculated the average entire duration for all relevant research projects. Here it was 6 weeks that produced the most beneficial results within groups. Drums were used for three out of the four projects that presented above average results. Once (Ashida, 2000) a small African drum was used for “drumming activity” at the start of every session. Each time a different participant was asked to perform with this instrument, although nobody in the experimental group was neither a professional drummer nor a musician. African drums were also used by another researcher (Chen et al., 2016). In addition, equipment also included one stereo, one electronic piano, two guitars, one set of hand glockenspiel, and other percussion instruments such as cymbals, tambourines, and xylophones. Finally, percussion instruments used in the third study (Lu et al., 2013) included hand bells, snare drums, a castanet, a tambourine, some claves, a triangle and wood blocks.

#### Total number of sessions – results

Continuing the analysis, we evaluated the total number of music intervention sessions. Apparently, this metric was dependent on the duration as well as frequency (“session frequency”) each intervention had. With one exception (Wang et al., 2011), where relevant data was missing, the number of sessions varied considerably. Only a single treatment session was used by three authors (Chan et al., 2010; Koelsch et al., 2010; Silverman, 2011), whereas 56 sessions (Castillo-Pérez et al., 2010) marked the opposite end of the scale. For 27 articles with available data, a combined average of 15 sessions was found. As far as the total number of sessions in an individual type of setting was concerned, above average results had a combined number of 13 (*SD* = 5) sessions, whereas the remaining six research works had 18 (*SD* = 8) interventions. The best results in a group setting showed an average of 17 sessions (*SD* = 15) and they were found in 7 scholarly publications. In comparison, we calculated 14 sessions in total for the remaining 7 articles.

#### Session frequency (i.e., sessions per week) – results

As described previously (Wheeler et al., 2003), the number of sessions can produce different results. Researchers, within our selection of 28 articles, used various approaches for their experiment, as far as the “session frequency” (i.e., number of sessions within a defined duration) was concerned. Pre-defined intervals ranged from once a week up to one time a day. Once (Choi et al., 2008), the article did mention the total number of sessions (*n* = 15) with a “frequency” of one to two times a week and a total intervention duration of 12 weeks. To be able to present an appropriate comparison of statistical data, a mean of 1.25 sessions per week was calculated. Besides two cases (Wang et al., 2011; Esfandiari and Mansouri, 2014) where no information was provided, the combined average session frequency for the remaining 26 articles was 2.89 (*SD* = 2.50) interventions per week. Usually sessions were held once a week.

#### Session- and research duration – vs. – [DSI] results in dependence of treatment setting

We further investigated if there was an association between therapy setting (individual or group), the length of a single session, and trial duration with regard to symptom improvement. Groups (Figure 3) showed better (i.e., above average) improvements in depression, if each session had an average duration of 60 min, and the mean length of treatment was 4–8 weeks.

In comparison, the two variables, session length and trial duration, had different effects for individual treatment approaches (Figure 3). Above average results were found for sessions lasting 30 min combined with a treatment duration between 4 and 8 weeks.

### Diagnostic measures – results of selected questionnaires

We discovered some distinctive features as well as certain similarities in our selection of 28 articles. They might be a guidance for future research projects and as such are presented in more detail in the subsections below.

### Beck depression inventory [BDI]

There are three versions of the BDI. The original [BDI] (Beck et al., 1961), followed by its first [BDI-I/-1A] (Beck et al., 1988) and second [BDI-II] revision (Beck et al., 1996). Beck used a novel approach to develop his inventory by writing down the verbal symptom description of his patients with depression and later sorted his notes according to intensity or severity.

#### Beck depression inventory [BDI] – results

The BDI^34^ (Beck et al., 1961, 1996) was the most widely used screening tool in our scholarly selection. It was used in eight trials, but we only selected 7^35^ studies for evaluating pre-post BDI scores. Once (Harmat et al., 2008), results were only provided for the experimental group, although an experimental control setting was described by the author. Twice (Harmat et al., 2008; Esfandiari and Mansouri, 2014) two experimental groups and one control group were reported. In one case (Esfandiari and Mansouri, 2014) two different music genres were used (“Light Pop & Heavy Rock”), and in another incident (Harmat et al., 2008) the second experimental group listened to an audiobook (“Music & Audiobook”). BDI baseline scores, that indicated a minimal^36^ to mild^37^ depression, were found in two articles (Gupta and Gupta, 2005; Harmat et al., 2008). Both authors reported for their experimental group a significant improvement of (BDI) depression scores. We calculated an overall average reduction of 2.72 (*SD* = 0.03). Moderate^38^ signs of depression, with BDI baseline scores that ranged from 18.66 (Albornoz, 2011) to 24.72 (Chen et al., 2016), were found twice. Music intervention improved BDI scores significantly, with an overall average reduction of 10.65 (*SD* = 3.63) for both articles mentioned above. For the respective control groups one author (Chen et al., 2016) reported non-significant pre-post changes, whereas the other researcher (Albornoz, 2011) described a significant^39^ reduction in the standard treatment group as well. The remaining three scholarly papers (Hendricks et al., 1999; Choi et al., 2008; Esfandiari and Mansouri, 2014) described participants with a severe^40^ depression, as confirmed by the initial (baseline) BDI results. One article (Esfandiari and Mansouri, 2014), of the three mentioned above, used one control and two experimental groups, who were treated with either “light” or “heavy” music. To be able to compare this work with the other studies one single baseline (31.75), post treatment (12.50), and pre-post difference score of 19.25 (*SD* = 2.47)^41^ was calculated (according to common statistical standards) for both experimental settings. Interestingly, the corresponding control sample showed a three-point increased BDI score (*p* > 0.05) and no decrease at any time. Continuing with the remaining articles, even bigger initial baseline BDI scores of 39.00 (*SD* = n/a) (Hendricks et al., 1999) and 49.30 (*SD* = 3.10) (Choi et al., 2008) were found. In addition, both authors reported a significant pre-post BDI score reduction^42^ for their experimental groups. Based on the published data it became evident that BDI scores improved significantly in each of the cases and this time an overall average reduction of 26.90 (*SD* = 9.59) was calculated. Once (Hendricks et al., 1999) a significantly reduced BDI pre-post score was also reported for the control setting, where participants received a cognitive-behavioral activities program as an alternative (non-music) intervention.

We compared all research projects that used the BDI questionnaire (Table 2). Higher baseline scores almost always led to comparatively bigger score reductions in those experimental groups, who received music intervention. Except for two articles (Hendricks et al., 1999; Albornoz, 2011), no significant improvements were found for control samples. For one of the above-mentioned exceptions (Hendricks et al., 1999) an alternative treatment (“*Cognitive-Behavioral” activities*) was provided, which might be a plausible explanation why those relatively young participants (all 14 or 15 years old) showed such reductions in BDI values. Nevertheless, it is also important to mention that the relevant experimental group improved to a greater extent (BDI_PRE_ − BDI_POST_ = 37.66) after treatment. As far as the other case (Albornoz, 2011) was concerned, no alternatives (i.e., other than basic or usual care) were offered, and thus no explanation had been established as to how the results could be explained.

**Table 2:** Comparison of BDI results.

**Authors [BDI]**	**Music Type/Genre (focus on)**	**Test-score [mean] Exp-Gr. Baseline**	**Test-score [mean] Exp-Gr. End/Post**	**Pre-Post Means Exp-Gr**.	**Score-Change in (%) Exp-Gr**.	***p*-Level Exp-Gr**.	**Pre-Post Means Con-Gr**.	***p*-Level Con-Gr**.
Albornoz, 2011	Percussion	18.66	10.58	[–]08.08	43.30	*p* < 0.005	[−]02.25	*p* < 0.05
Chen et al., 2016	Percussion	24.72	11.51	[–]13.21	53.44	*p* < 0.01	[−]03.58	*p* > 0.05
Choi et al., 2008	Korean	49.30	25.50	[–]23.80	48.28	*p* < 0.001	[−]02.90	*p* > 0.05
Esfandiari and Mansouri, 2014	Pop & Rock	31.75	12.50	[–]19.25	60.63	*p* < 0.05	[+]03.00	*p* > 0.05
Gupta and Gupta, 2005	Indian Flute	08.94	06.24	[–]02.70	30.20	*p* < 0.001	[−]00.27	*p* > 0.05
Harmat et al., 2008	Classical	05.40	02.66	[–]02.74	50.74	*p* < 0.05	n/a	*p* > 0.05
Hendricks et al., 1999	Relaxing	39.00	01.34	[–]37.66	96.56	*p* < 0.05	[−]15.30	*p* < 0.05
Silverman, 2011	Songwriting	–	18.79	[–]00.00	–	*p* > 0.05	[−]01.49	*p* > 0.05

### Geriatric depression scale [GDS-15/-30]

The original *Geriatric Depression Scale* [GDS-30] (Yesavage et al., 1983) includes 30 questions (Hanser and Thompson, 1994; Chan et al., 2009; Guétin et al., 2009a) and its shorter equivalent [GDS-15] (Yesavage and Sheikh, 1986) contains 15 items (Chan et al., 2010, 2012; Verrusio et al., 2014).

#### Geriatric depression scale [GDS-15/-30] – results

A more precise analysis of results was also done for the Geriatric Depression Scale (GDS-15/-30) scores. As already suggested by its name, all 223 participants were elderly. Because both GDS versions are based on the same questionnaire, we combined scores of the long (i.e., GDS-30) with the short (i.e., GDS-15) test version and found a total of 223 participants in six articles (e.g., Chan et al., 2009; Verrusio et al., 2014). A possible bias could be prevented because tests were evenly distributed in number, and with respect to higher GDS-30 as well as lower GDS-15 scores, calculations were adapted accordingly. Taking a closer look at the GDS-15/-30 results (Table 3), some similarities could be found for the most successful (all *p* ≤ 0.01) four research articles (Chan et al., 2009, 2010; Guétin et al., 2009a; Verrusio et al., 2014). All of them used and mainly focused on classical compositions as far as their music title selection was concerned. The average reduction in depression as measured by the GDS-15/-30 depression scores was 43% (−42.62%; *SD* = 6.24%). In comparison, every one of the remaining four research projects (Hanser and Thompson, 1994; Ashida, 2000; Han et al., 2011; Chan et al., 2012) also presented significant results, albeit not as good as the above-mentioned (all *p* ≤ 0.05). Interestingly, as far as music genres were concerned, the focus of these less successful projects was rhythmic drumming in two cases (Ashida, 2000; Han et al., 2011). For the remaining two (Hanser and Thompson, 1994; Chan et al., 2012) primarily relaxing, slow paced titles^43^ were selected as intervention.

**Table 3:** Comparison of GDS-15/-30 Results (^*^)GDS-15, (^**^)GDS-30.

**Authors [BDI]**	**Music Type/Genre (focus on)**	**Test-score [mean] Exp-Gr. Baseline**	**Test-score [mean] Exp-Gr. End/Post**	**Pre-Post Means Exp-Gr**.	**Score-change in [%] Exp-Gr**.	***p*-Level Exp-Gr**.	**Pre-Post Means Con-Gr**.	***p*-Level Con-Gr**.
Chan et al., 2010^*^	Relaxing	04.10	02.10	[–]02.00	48.78	*p* < 0.001	[+]00.20	*p* > 0.05
Chan et al., 2012^*^	Relaxing	04.17	01.38	[–]02.79	66.91	*p* < 0.05	[–]00.08	*p* > 0.05
Verrusio et al., 2014^*^	Jazz/Classic	08.50	05.50	[–]03.00	35.29	*p* < 0.01	[–]00.40	*p* > 0.05
Chan et al., 2009^**^	Jazz/Classic	13.10	07.90	[–]05.20	39.69	*p* < 0.005	[+]02.40	*p* = 0.007
Guétin et al., 2009a^**^	Jazz/Classic	16.70	08.90	[–]07.80	46.71	*p* < 0.01	[–]00.60	*p* > 0.05
Hanser and Thompson, 1994^**^	Harp & PMR	17.45	10.00	[–]07.45	42.69	*p* < 0.05	[+]00.90	*p* > 0.05

### Other diagnostic measures for depression^44^ – results^45^

Several times, additional questionnaires were used to measure changes in the severity of depression.

Researchers performed those surveys (Table 4) in addition to their “main” depression questionnaire. Please refer to our Supplementary Material for a more comprehensive test description.

**Table 4:** Additional tests, conducted by researchers within our article selection for investigating changes in depression.

**Test-Name**	**Abbrevations**	**Creator(s)**	**Used by**
Calgary Depression (rating) Scale for Schizophrenia	CDSS	Addington et al., 1990	Lu et al., 2013
Center for Epidemiological Studies Depression Scale	CES-D	Radloff, 1977	Schwantes and Mckinney, 2010
Cornell Scale for Depression in Dementia	CSDD	Alexopoulos et al., 1988	Ashida, 2000
Edinburgh Postnatal Depression Scale	EPDS	Cox et al., 1987	Chang et al., 2008
Hospital Anxiety and Depression Scale - Depression-Subscale	HAD(S)-D	Zigmond and Snaith, 1983	Guétin et al., 2009b; Castillo-Pérez et al., 2010; Fancourt et al., 2016
Hamilton Rating Scale for Depression	HAM-D (=) HRSD	Hamilton, 1960	Albornoz, 2011
Montgomery-Åsberg Depression Rating Scale	MADRS	Montgomery and Asberg, 1979	Deshmukh et al., 2009; Erkkilä et al., 2011
Perception (“Profile”) of Mood States (short 35-item version)-Depression Sub-Scale	POMS(-SF)_D_	Curran et al., 1995	Koelsch et al., 2010
Revised Memory and Behavioral Problems Checklist-Depression	RMBPC_D_	Johnson et al., 2001	Han et al., 2011
Self-Rating Depression Scale	SDS	Zung, 1965	Hsu and Lai, 2004; Kim et al., 2006; Castillo-Pérez et al., 2010; Wang et al., 2011

### Diagnostic measures for pathologies other than depression – results

In many instances, additional questionnaires were used (Table 5)^49^ to measure symptoms other than depression (e.g., Anxiety is known to be one of the most common depression comorbidities, Sartorius et al., 1996; Bradt et al., 2013; Tiller, 2013). Eight^46^ researchers concentrated their investigation entirely on depression, and thus only performed questionnaires related to this pathology. In comparison, most of the remaining studies measured additional pathologies, with some of them known to be often associated comorbidities with depressive symptoms. However, because these topics were not the focus of this review, we won't discuss them here in detail. A much more detailed representation is available in the Supplementary Table. Please refer to the original studies for a more comprehensive test description.

**Table 5:** Additional tests, conducted by researchers within our selection for investigating changes in other pathologies.

**Area**	**Questionnaire-Name**	**Abbrevations**	**Creator(s)**	**Authors**
Anxiety	Apparent Emotion Scale-Anxiety	AES_A_	Lawton et al., 1999	Han et al., 2011
Anxiety	Four Factor Anxiety Inventory	FFAI	Gupta and Gupta, 1998	Gupta and Gupta, 2005
Anxiety	Hamilton's Anxiety Rating Scale	HAM-A (=) HAS	Hamilton, 1959	Guétin et al., 2009b; Verrusio et al., 2014
Anxiety	Hospital Anxiety and Depression Scale-Anxiety-Subscale	HAD(S)-A	Zigmond and Snaith, 1983	Guétin et al., 2009b; Erkkilä et al., 2011; Fancourt et al., 2016
Anxiety	State-Trait Anxiety Inventory	STAI	Spielberger et al., 1983	Gupta and Gupta, 2005; Chang et al., 2008; Choi et al., 2008; Chen et al., 2016
Confidence and Self-esteem	Rosenberg Self-Esteem Inventory	RSI (=) SEI	Rosenberg, 1965	Hanser and Thompson, 1994; Chen et al., 2016
Psychopathology	Symptom Checklist-90	SCL-90	Derogatis, 1975	Wang et al., 2011
Sleep	Epworth Sleepiness Scale	ESS	Johns, 1991	Harmat et al., 2008
Sleep	Pittsburgh Sleep Quality Index	PSQI	Buysse et al., 1989	Harmat et al., 2008; Deshmukh et al., 2009; Chan et al., 2010
Likert-Scale	Self-Assessment Manikins	SAMs	Bradley and Lang, 1994	Koelsch et al., 2010
Likert-Scale	Seven-point Likert scale	—	Silverman, 2011	Silverman, 2011
Likert-Scale	Toronto Alexithymia Scale	TAS-26	Kupfer et al., 2001	Koelsch et al., 2010
Symptom	Brief Symptom Inventory-General Severity Index	BSI-GSI	Derogatis and Spencer, 1982; Derogatis and Melisaratos, 1983	Hanser and Thompson, 1994
Symptom	Brief Symptom Inventory-18	BSI-18	Derogatis, 2000	Schwantes and Mckinney, 2010
Symptom	Positive and Negative Syndrome Scale	PANSS	Kay et al., 1987	Lu et al., 2013
Comorbidity	Comorbidity Index	CInd	Charlson et al., 1987	Verrusio et al., 2014
Illness	Cumulative Illness Rating Scale	CIRS	Linn et al., 1968	Verrusio et al., 2014

## Discussion, conclusion and further thoughts

Depression often reduces participation in social activities. It also has an impact on reliability or stamina at daily work and may even result in a greater susceptibility to diseases. Music can be considered an emerging treatment option for mood disorders that has not yet been explored to its full potential. To the best of our knowledge, there were only very few meta-analyses, or systematic reviews of randomized controlled trials available that generated the amount of statistical data, which we presented here.

Certain individual-specific attributes of music are recognizable, when the medium of music is decomposed (Durkin, 2014)^47^ into its components. Numerous researchers reported the beneficial effects of music, such as strengthening awareness and sensitiveness for positive emotions (Croom, 2012), or improvement of psychiatric symptoms (Nizamie and Tikka, 2014). Group drumming, for example, helped soldiers to deal with their traumatic experiences, while they were in the process of recovery (Bensimon et al., 2008). However, we have concentrated our focus of interest on patients diagnosed with clinical depression, one of the most serious and frequent mental disorders worldwide.

In this review we examined whether, and to what extent, music intervention could significantly affect the emotional state of people living with depression. Our primary objective was to accurately identify, select, and analyze up-to-date research literature, which utilized music as intervention to treat participants with depressive symptoms. After a multi-stage review process, a total of 1.810 participants in 28 scholarly papers met our inclusion criteria and were finally selected for further investigations about the effectiveness music had to treat their depression. Both, quantitative as well as qualitative empirical approaches were performed to interpret the data obtained from those original research papers. To consider the different methods researchers used, we presented a detailed illustration of approaches and evaluated them during our investigation process.

Interventions included, for example, various instrumental or vocal versions of classical compositions, Jazz, world music, and meditative songs to name just a few genres. Classical music (Classical or Baroque period) for treatment was used in nine articles. Notable composers were W.A. Mozart, L. v. Beethoven and J. S. Bach. Jazz was used five times for intervention. Vernon Duke (Title: “April in Paris”), M. Greger (Title: “Up to Date”), or Louis Armstrong (Title: “St. Louis Blues”) are some of the featured artists. The third major genre researchers used for their experimental groups was percussion and drumming-based music.

Significant criteria were complete trial duration, amount of intervention sessions, age distribution within participants, and individual or group setting. We compared passive listening to recorded music (e.g., CD), with active experiencing of live music (e.g., singing, improvising with instruments). Furthermore, the analysis of similar studies has enhanced and complemented our work. Previous studies indicated positive effects of music on emotions and anxiety, what we tried to confirm in more detail. The length of an entire music treatment procedure was suspected to be an important element for reducing symptoms of depression. A longer treatment duration of 7 weeks for an individual, compared to nearly 6 weeks in a group setting led to better (i.e., above average) outcomes. Although a difference was discovered, 1 week was not enough to draw further conclusions for each and every project. As far as intervals between sessions were concerned, we found no differences between those research articles that were among the best, compared to the remaining experimental designs. Consequently no trend was becoming apparent, favoring one over the others. We further investigated if there was any association between an individual or a group setting, if the length of a single session and trial duration were compared with regard to symptom improvement. Groups showed better improvements in depression, if each session had an average duration of 60 min, and a treatment between 4 and 8 weeks long. In comparison, the two variables, session length and trial duration, had different effects for individual treatment approaches. Above average results were found for sessions lasting 30 min combined with a treatment duration between 4 and 8 weeks. Furthermore, results were compared according to age groups (“young,” “medium,” and “elderly”). Overall, elderly people benefitted in particular from this kind of non-invasive treatment. During, but mainly after completion of music-driven interventions, positive effects became apparent. Those included primarily social aspects of life (e.g., an increased motivation to participate in life again), as well as concerned participants' psychological status (e.g., a strengthened self-confidence, an improved resilience to withstand stress).

We described similarities, the integration of different music intervention approaches had on participants in experimental vs. control groups, who received an alternative, or no additional treatment at all. Additional questionnaires confirmed further improvements regarding confidence, self-esteem and motivation. Trends in the improvement of frequently occurring comorbidities (e.g., anxiety, sleeping disorders, confidence and self-esteem)^48^, associated with depression, were also discussed briefly, and showed promising outcomes after intervention as well. Particularly anxiety (Sartorius et al., 1996; Tiller, 2013) is known to be a common burden, many patients with mood disorders are additionally affected with. Interpreted as manifestation of fear, anxiety is a basic feeling in situations that are regarded as threatening. Triggers can be expected threats such as physical integrity, self-esteem or self-image. Unfortunately, researchers merely distinguished between “anxiety disorder” (i.e., mildly exceeded anxiety) and the physiological reaction. Also, the question should be raised if the response to music differs if patients are suffering from both, depression and anxiety. Sleep quality in combination with symptoms of depression (Mayers and Baldwin, 2006) raised the question, whether sleep disturbances lead to depression or, vice versa, depression was responsible for a reduced quantity of sleep instead. Most studies used questionnaires that were based on self-assessment. However, it is unclear whether this approach is sufficiently valid and reliable enough to diagnose changes regarding to symptom improvement. Future approaches should not solely rely on questionnaires, but rather add measurements of physiological body reactions (e.g., skin conductance, heart and respiratory rate, or AEP's via an EEG) for more objectivity.

The way auditory stimuli were presented, also raised some additional questions. We found that for individual intervention most of the times headphones were used. For a group setting speakers were the number one choice instead. For elderly participants, a different sensitivity for music perception was a concern, when music was presented directly through headphones. Headphones add at least some isolation from background noises (i.e., able to reduce noise disturbances and surround-soundings). Another concern was that most of the time a certified hearing test was not used. Although, a tendency toward a reduction in the ability to hear higher frequencies is quite common with an increased age, there might still be substantial differences between participants.

Two authors (Deshmukh et al., 2009; Silverman, 2011) reported that participants within their respective music intervention group, did not present a significant reduction of depression. Those two had almost nothing in common^49^ and were not investigated further.

Control groups, who received an alternative (“non-music”) intervention, were found in nine research articles. Significant reduction of depression in corresponding control (“non-music intervention”) groups was reported by two authors (Hendricks et al., 1999; Albornoz, 2011). In one instance (Albornoz, 2011) the relevant participants received only standard care, but in the other case (Hendricks et al., 1999) an alternative treatment (Cognitive-Behavioral activities) was reported. Medical conceptions are in a constant state of change. To achieve improvements in areas of disease prevention and treatment, psychology is increasingly associated with clinical medicine and general practitioners. Under the guidance of an experienced music therapist, the patient receives a multimodal and very structured treatment approach. That is the reason why we can find specialists for music therapy in fields other than psychosomatics or psychiatry today. Examples are internal medicine departments and almost all rehabilitation centers. The acoustic and musical environment literally opens a portal to our unconscious mind. Music therapy often comes into play when other forms of treatment are not effective enough or fail completely.

Music connects us to the time when we only had preverbal communication skills (Hwang and Hughes, 2000; Graham, 2004; i.e., communication before a fully functioning language is developed; e.g., infants or children with autism spectrum disorder), without being dependent on language. Although board-certified music therapy is undeniable the most regulated, developed and professional variant, this should not hinder health professionals and researchers from other areas in the execution of their own projects using music-based interventions. The only thing they should be very precise about, is the way they define their work. Within our selection of articles the expression music therapy was used sometimes, although a more detailed description or specific information was neither published nor available upon our request. In those cases, the term “music therapy” should not be used, but instead music medicine or some of the alternatives mentioned in this manuscript (e.g., therapy with music, music for treatment). This way many obstacles as well as misunderstandings can be prevented in the first place, but high-quality research is still produced. Also, it is very important that researchers contemplate and report the details of the music intervention that they use. For example, they should report whether the music is researcher-selected or participant-selected, the specific tracks they used, the delivery method (speakers, headphones), and any other relevant details.

Encouraged by the promising potential of music as an intervention (Kemper and Danhauer, 2005), we pursued our ambitious goal to contribute knowledge that provides help for the affected individuals, both the patients themselves as well as their nearest relatives. Furthermore, we wanted to provide detailed information about each randomized controlled study, and therefore made all our data available, so others may benefit for their potential upcoming research project. The overall outcome of our analysis, with all significant effects considered, produced highly convincing results that music is a potential treatment option, to improve depression symptoms and quality of life across many age groups. We hope that our results provide some support for future concepts.

## Author contributions

DL (Substantial contributor who meets all four authorship criteria): (1) Project idea, article concept and design, as well as planning the timeline, substantially involved in the data, material, and article acquisition, (2) mainly responsible for drafting, writing, and revising the review article, (3) responsible for selecting and final approving of the scholarly publication, (4) agreed and is accountable for all aspects connected to the work. TH (Substantial contributor who meets all four authorship criteria): (1) Substantial help with the concept and design, substantially contributed to the article and material acquisition, (2) substantially contributed to the project by drafting and revising the review article, (3) responsible for final approval of the scholarly publication, (4) agreed and is accountable for all aspects connected to the work.

### Conflict of interest statement

The authors declare that the research was conducted in the absence of any commercial or financial relationships that could be construed as a potential conflict of interest.

## Footnotes

^1^Participants in the two music groups (standard or patterning music) showed an increased belief in their personal power as well as a reduction in pain, depression and disability, compared to the relevant control group. The two experimental groups listened to 1 h of music each day for 7 days in a row.

^2^Official definition of the American Music Therapy Association [AMTA] http://www.musictherapy.org/about/quotes/

^3^Clinical speciality areas; Diagnostic, Treatment, and Therapeutic procedures, approaches, tools; Disorders; Age groups; Scientific; Country-specific; Musical Aspects; Recording hardware and equipment; Literature Genre; Publication type or medium; Year of publication; Number of authors.

^4^Boolean Operators for searching databases: Concept explained by the Massachusetts Institute of Technology [MIT].

^5^Our preference was an experimental-control setting, but unfortunately three authors (Ashida, 2000; Guétin et al., 2009b; Schwantes and Mckinney, 2010) did not use a control sample.

^6^AMSTAR (Shea et al., 2007)–Further Info & AMSTAR online calculator: https://amstar.ca/Amstar_Checklist.php; National Collaborating Centre for Methods and Tools (NCCMT): http://www.nccmt.ca/resources/search/97 Questions included in the AMSTAR-Checklist (Shea et al., 2007) are: (I) Was an “a priori” design provided? (II) Was there duplicate study selection and data extraction? (III) Was a comprehensive literature search performed? (IV) Was the status of publication (i.e. gray literature) used as an inclusion criterion? (V) Was a list of studies (included and excluded) provided? (VI) Were the characteristics of the included studies provided? (VII) Was the scientific quality of the included studies assessed and documented? (VIII) Was the scientific quality of the included studies used appropriately in formulating conclusions? (IX) Were the methods used to combine the findings of studies appropriate? (X) Was the likelihood of publication bias assessed? (XI) Was any conflict of interest included?

^7^In our Supplementary Table (“Complete Display of Statistical Data”), DSI was referred to as “Change [%].”

^8^A much More Detailed Representation of Demographics is Available in the Supplementary Table (Appendix-B).

^9^9DSI: Depression Score Improvement stands for the mean difference between the pre-test and post-test results (i.e., score changes) in percent. Please refer to the supplementary materials for additional information.

^10^Music interventions: Individual setting (Chang et al., 2008); Group setting (Esfandiari and Mansouri, 2014).

^11^One [MT] music-therapy article (Silverman, 2011) was not used for comparison and calculations because the relevant data was unavailable.

^12^Djembe is based on the expression “anke djé, anke bé” which roughly translates as “everyone should come together in peace and harmony.”

^13^12-bar Blues: Traditional Blues pattern that is 12 measures long. This chord progression is also used for many other music genres and quite popular in pop-music.

^14^Ambiguity of the term “classical” music: In our review, this term refers to “Western Art Music” and thus includes, but is not limited to the “Classical” music period. Most of the time we used this term for music from the Baroque (1600–1750), Classical (1750–1820), and Romantic (1804–1910) period.

^15^Within percussion groups various types of drums presented the instrument of choice most of the time.

^16^Eight out of nine articles because in on case (Castillo-Pérez et al., 2010) scores were missing. The remaining were: Hsu and Lai, 2004; Chang et al., 2008; Harmat et al., 2008; Chan et al., 2009, 2010; Guétin et al., 2009a; Koelsch et al., 2010; Verrusio et al., 2014.

^17^Average: Arithmetic mean of all score-changes in [%] for a defined selection (e.g., classical music). Example: We calculated the score-change in [%] for each of the eight experimental groups that received classical music as intervention. In this case the arithmetic mean (DSI_Clas_) was 39.98% (i.e., average). Then every individual score can be compared to this average. If it was above, we called it “above average”.

^18^Percussion music (drumming): Ashida, 2000; Choi et al., 2008; Schwantes and Mckinney, 2010; Albornoz, 2011; Erkkilä et al., 2011; Han et al., 2011; Lu et al., 2013; Chen et al., 2016; Fancourt et al., 2016.

^19^Jazz: Chan et al., 2009, 2010; Guétin et al., 2009a; Koelsch et al., 2010; Verrusio et al., 2014.

^20^In most cases there was no further categorization between different musical sub-genres of Jazz.

^21^Greatest: Best in terms of depression score improvement (DSI) (i.e., pre-post score reduction in percent) with Jazz as intervention.

^22^Raga: Classification system for music that originated during the eleventh century in Asia (mainly India).

^23^Setting was always: Experimental group received music as intervention, and the corresponding control group received an (non-music) alternative.

^24^For example, if elderly people lived in a retirement home, a standard daily routine or common everyday activities were seen as usual or regular treatment. If, on the other hand, a resting period (e.g., Chan et al., 2012) was carried out simultaneously, this was interpreted as an (“non-music”) alternative.

^25^In all three of his articles within our selection (Chan et al., 2009, 2010, 2012) participants were instructed to rest.

^26^Pharmacotherapy treatment included SSRI (Paroxetine 20mg/die), NaSSA (Mirtazapine 30 mg/die), and Benzodiazepine (Alprazolam).

^27^As already described above, the other individual setting (Guétin Soua, et al., 2009) with pre-post results of *p* > 0.05 was still counted as significant.

^28^Information regarding the duration for one group session was unavailable in two articles (Hendricks et al., 1999; Wang et al., 2011).

^29^Hanser and Thompson, 1994; Hsu and Lai, 2004; Harmat et al., 2008; Chan et al., 2009, 2010, 2012; Guétin et al., 2009a; Erkkilä et al., 2011.

^30^Average DSI for all 13 articles that used an individual (^*^Ind) treatment as intervention was 36.50%.

^31^Gupta and Gupta, 2005; Kim et al., 2006; Chang et al., 2008; Deshmukh et al., 2009; Guétin et al., 2009b.

^32^Once (Esfandiari and Mansouri, 2014) the relevant score was unavailable.

^33^Once (Wang et al., 2011) the relevant score was unavailable.

^34^BDI: Original BDI from1961; (1st) Revision (=) BDI-I or BDI-1A from 1978; (2nd) Revision (=) BDI-II from 1996.

^35^BDI-scores were measured only once (Silverman, 2011), either at the end (experimental group), or at the beginning (control group) and thus was excluded for this calculation.

^36^Minimal depression: BDI-I (= BDI-1A) score (=) 00–09; BDI-II score (=) 00–13.

^37^Mild depression: BDI-I (= BDI-1A) score (=) 10–18; BDI-II score (=) 14–19.

^38^Moderate depression: BDI-I (= BDI-1A) score (=) 19–29; BDI-II score (=) 20–28.

^39^Albornoz (2011) found in both groups a significant reduction for BDI scores albeit to a significantly greater extent in the experimental (−8.08; *p* < 0.01) than in the control (−2.25; *p* < 0.05) setting.

^40^Severe depression: BDI-I (=BDI-1A) score (=) 30–63; BDI-II score (=) 29–63.

^41^Pre-post difference: experimental (1) “light” music (=) 17.50; experimental (2) “heavy” music (=) 21.00 (both *p* < 0.05 within groups) (Esfandiari and Mansouri, 2014).

^42^Average pre-post BDI reduction of −30.73 (*SD* = 9.80) combined (Hendricks et al., 1999; Choi et al., 2008).

^43^One author (Chan et al., 2012) limited his selection to slow music (60–80 beats per minute). The other researcher (Hanser and Thompson, 1994) also used some “energetic” or “empowering” titles, but mainly concentrated on relaxing compositions.

^44^for a reference “intervention review” about music therapy for depression see: maratos et al. (2008).

^45^Every available test-result (Pre-/Post-Scores for experimental/control) can be found in our Supplementary Table 12.

^46^Hendricks et al., 1999; Ashida, 2000; Hsu and Lai, 2004; Kim et al., 2006; Chan et al., 2009, 2012; Castillo-Pérez et al., 2010; Albornoz, 2011.

^47^We used the metaphor “decomposed” based on the inspiring book by Andrew Durkin (“Decomposition: A Music Manifesto”), who refers to it “as a way…to demythologize music without demeaning it” (Review by Madison Heying).

^48^A complete list, with all results we could extract, can be found in the Supplementary Table.

^49^Music Therapy; Duration 90min./session; Session Frequency 7x/week; Raagas Music (Deshmukh et al., 2009).
