# Supplementary material for: Reviewing the Effectiveness of Music Interventions in Treating Depression
Source: Front Psychol. 2017 Jul 7;8:1109. doi: 10.3389/fpsyg.2017.01109 (PMC5500733; doi:10.3389/fpsyg.2017.01109)
Supplement: Supplementary file 1 [file DataSheet1.pdf]

AUTHOR	YEAR	PARTICIPANTS [no.] ALL	PARTICIPANTS [no.] EXP.	PARTICIPANTS [no.] CONT.	AGE GROUP	AGE [y] ALL Range	AGE [y] ALL Mean (SD)	AGE [y] Experimental Range	AGE [y] Experimental Mean (SD)	AGE [y] Control Range	AGE [y] Control Mean (SD)	GENDER ALL Male	GENDER ALL Female	GENDER Experimental Male	GENDER Experimental Female	GENDER Control Male	GENDER Control Female
**Albornoz**	**2011**	24	12	12	Medium	16-60	(–?–)	16-60	(–?–)	16-60	(–?–)	24	**0**	12	**0**	12	**0**
**Ashida**	**2000**	20	20	**0**	Elderly	73-94	86.20 (–?–)	73-94	86.20 (–?–)	(–)	(–)	3	17	3	17	**0**	**0**
**Castillo-Pérez** et al.	**2010**	79	41	38	Medium	25-60	42.50 (–?–)	25-60	(–?–)	25-60	(–?–)	14	65	8	33	6	32
**Chan** et al.	**2009**	47	23	24	Elderly	60-80+	(–?–)	60-80+	(–?–)	60-80+	(–?–)	21	26	10	13	11	13
**Chan** et al.	**2010**	42	21	21	Elderly	60-80+	(–?–)	60-80+	(–?–)	65-80+	(–?–)	19	23	9	12	10	11
**Chan** et al.	**2012**	50	24	26	Elderly	55-75+	(–?–)	55-75+	(–?–)	55-75+	(–?–)	18	32	9	15	9	17
**Chang** et al.	**2008**	236	116	120	Medium	20-41	30.02 (03.66)	24-41	30.48 (–?–)	20-39	29.58 (–?–)	**0**	236	**0**	116	**0**	120
**Chen** et al.	**2016**	200	100	100	Medium	18-57	35.50 (09.95)	18-57	35.29 (09.83)	18-57	35.75 (10.07)	200	**0**	100	**0**	100	**0**
**Choi** et al.	**2008**	26	13	13	Medium	(–?–)	36.15 (10.20)	(–?–)	37.40 (10.90)	(–?–)	34.90 (09.50)	12	14	7	6	5	8
**Deshmukh** et al.	**2009**	50	25	25	Medium	21-51	33.00 (00.60)	(–?–)	(–?–)	(–?–)	(–?–)	24	26	(–?–)	(–?–)	(–?–)	(–?–)
**Erkkilä** et al.	**2011**	79	33	46	Medium	18-50	35.65 (–?–)	18-50	35.80 (09.00)	18-50	35.50 (10.50)	17	62	8	25	9	37
**Esfandiari** and **Mansouri**	**2014**	30	7_{LIGHT}	14	Young	18-30	(–?–)	18-30	(–?–)	18-30	(–?–)	**0**	30	**0**	7_{LIGHT}	**0**	14
			9_{HEAVY}												9_{HEAVY}		
**Fancourt** et al.	**2016**	45	30	15	Medium	(–?–)	53.54 (13.90)	(–?–)	55.07 (13.00)	(–?–)	52.00 (14.70)	8	37	7	23	1	14
**Guétin, Portet** et al.	**2009**	30	15	15	Elderly	74-95	(–?–)	75-93	85.20 (06.00)	74-95	86.90 (05.20)	8	22	2	13	6	9
**Guétin, Soua** et al.	**2009**	13	13	**0**	Medium	25-50	31.10 (09.30)	25-50	31.10 (09.30)	(–)	(–)	3	10	3	10	**0**	**0**
**Gupta** and Gupta	**2005**	80	40	40	Young	19-24	21.26 (02.62)	19-24	21.55 (01.47)	19-24	20.98 (03.40)	80	**0**	40	**0**	40	**0**
**Han** et al.	**2011**	43	28	15	Elderly	60-80+	78.30 (08.15)	60-80+	78.10 (07.20)	65-80+	78.50 (09.10)	12	31	7	21	5	10
**Hanser** and **Thompson**	**1994**	30	$10_{ThVisit}$	10	Elderly	61-86	67.90 (–?–)	61-86	(–?–)	61-86	(–?–)	7	23	(–?–)$_{Th.Vi}$	(–?–)$_{Th.Vi}$	(–?–)	(–?–)
			10_{PhCall}											(–?–)$_{Ph.C}$	(–?–)$_{Ph.C}$	(–?–)	(–?–)
**Harmat** et al.	**2008**	94	35_{Music}	29	Young	19-28	22.60 (02.83)	19-(?)	(–?–)	19-(?)	(–?–)	21	73	(–?–)$_{Musi}$	(–?–)$_{Musi}$	(–?–)	(–?–)
			30_{AuBo}					19-(?)	(–?–)					(–?–)$_{AuB}$	(–?–)$_{AuB}$	(–?–)	(–?–)
**Hendricks** et al.	**1999**	19	9	10	Young	14-15	(–?–)	14-15	(–?–)	14-15	(–?–)	2	17	(–?–)	(–?–)	(–?–)	(–?–)
**Hsu** and **Lai**	**2004**	54	27	27	Medium	18-73	37.00 (12.50)	(–?–)	(–?–)	(–?–)	(–?–)	(–?–)	(–?–)	(–?–)	(–?–)	(–?–)	(–?–)
**Kim** et al.	**2006**	36	18	18	Medium	25-60	(–?–)	25-60	(–?–)	25-60	(–?–)	18	18	9	9	9	9
**Koelsch** et al.	**2010**	154	81	73	Young	18-31	24.60 (03.00)	19-31	24.40 (–?–)	18-31	24.90 (–?–)	76	78	39	42	37	36
**Lu** et al.	**2013**	80	38	42	Medium	35-65	52.02 (–?–)	(–?–)	51.66 (07.46)	(–?–)	52.35 (07.87)	59	21	27	11	32	10
**Schwantes** and **McKinney**	**2010**	5	5	**0**	Medium	21-53	**n/a**	21-53	**n/a**	(–)	(–)	5	**0**	5	**0**	**0**	**0**
**Silverman**	**2011**	140	69	71	Medium	(–?–)	43.20 (12.24)	(–?–)	43.33 (13.08)	(–?–)	43.07 (11.40)	70	70	33	36	37	34
**Verrusio** et al.	**2014**	24	12	12	Elderly	(–?–)	75.50 (07.40)	(–?–)	74.80 (08.00)	(–?–)	76.10 (07.10)	11	13	7	5	4	8
**Wang** et al.	**2011**	80	40	40	Young	18-30	19.35 (01.68)	18-30	(–?–)	18-30	(–?–)	(–?–)	(–?–)	(–?–)	(–?–)	(–?–)	(–?–)

AUTHOR	YEAR	MUSIC INTERVENTION: INS TRU MEN	Voca & Sing	LIVE	MUSIC TYPE	REC	Spea kers	Hea Pho.	Setting	SESSIONS (TIMETABLE): [MT] Music Therapy [MM] Music Medicine	1 Session Duration	Total № Sessions	Session-ƒrequen.	ENTIRE Research Duration
**Albornoz**	**2011**	(+)	(–)	(+)	**Drumming**, Percussion Improvisation	(–)	(–)	(–)	Group	M. Medicine	120 min.	012x	1x/week	12w 0d
**Ashida**	**2000**	(+)	(+)	(+)	**Drumming**, 1890-1930 Music	(–)	(–)	(–)	Group	M. Medicine	043 min.	005x	5x/week	0w 5d
**Castillo-Pérez** et al.	**2010**	(+)	(–)	(–)	**Classical:** Classic Period: (Bach et al.) Baroque Period: (Mozart et al.)	(+)	(+)	(+)	**6x**Group and **1x**Individ.	M. Medicine	050 min.	056x	7x/week	8w 0d
**Chan** et al.	**2009**	(+)	(+)	(–)	**Classical**, **Jazz**, Asian	(+)	(+)	(–?–)	Individual	M. Medicine	030 min.	016x	4x/week	4w 0d
**Chan** et al.	**2010**	(+)	(–)	(–)	**Classical**, **Jazz**, Relax-Music, Asian	(+)	(–)	(+)	Individual	M. Medicine	030 min.	004x	1x/week	4w 0d
**Chan** et al.	**2012**	(+)	(+)	(–)	Rhythmic (slow), Asian, Western	(+)	(+)	(+)	Individual	M. Medicine	030 min.	008x	1x/week	8w 0d
**Chang** et al.	**2008**	(+)	(–)	(–)	**Classical**, **Lullabies**, Nature	(+)	(+)	(–)	Individual	M. Medicine	030 min.	014x	7x/week	2w 0d
**Chen** et al.	**2016**	(+)	(+)	(+)	**Drumming**, Piano (playing)	(+)	(+)	(–)	Group	M. Medicine	090 min.	020x	2x/week	10w 0d
**Choi** et al.	**2008**	(+)	(+)	(+)	**Drumming**, Relax-Music, Singing	(+)	(+)	(–)	Group	**M. Therapy**	060 min.	015x	1-2x/week	12w 0d
**Deshmukh** et al.	**2009**	(+)	(–)	(–)	**Raagas**-Music Selection	(+)	(+)	(–)	Individual	M. Medicine	090 min.	028x	7x/week	4w 0d
**Erkkilä** et al.	**2011**	(+)	(–)	(+)	**Drumming** (Djembe Drum)	(–)	(–)	(–)	Individual	**M. Therapy**	060 min.	020x	2x/week	10w 0d
**Esfandiari** and **Mansouri**	**2014**	(+)	(–)	(–)	Light **POP** Music	(+)	(+)	(–)	Group	M. Medicine	045 min.	014x	(–?–)	(–?–)
					Heavy Hard **ROCK**									
**Fancourt** et al.	**2016**	(+)	(–)	(+)	**Drumming**	(–)	(–)	(–)	Group	M. Medicine	090 min.	010x	1x/week	10w 0d
**Guétin, Portet** et al.	**2009**	(+)	(+)	(–)	**Classical**, **Jazz**, World-Music	(+)	(–)	(+)	Individual	M. Medicine	020 min.	016x	1x/week	16w 0d
**Guétin, Soua** et al.	**2009**	(+)	(+)	(+)	Relax-Music	(+)	(–)	(+)	Individual	M. Medicine	060 min.	020x	1x/week	20w 0d
**Gupta** and Gupta	**2005**	(–)	(+)	(–)	Indian **Flute** (“Raga Desi-Todi”)	(+)	(–)	(+)	Individual	M. Medicine	030 min.	020x	7x/week	2w 6d
**Han** et al.	**2011**	(+)	(+)	(+)	**Drumming**, Singing, Dancing, Improvisation with various Instruments	(+)	(+)	(–)	Group	**M. Therapy**	120 min.	008x	1x/week	8w 0d
**Hanser** and **Thompson**	**1994**	(+)	(+)	(–)	Relax-Music, Improvisational Harp Music, PMR (spoken cues)	(+)	(+)	(–)	Individual	**M. Therapy**	033 min.	008x	1x/week	8w 0d
**Harmat** et al.	**2008**	(+)	(+)	(–)	**Classical** Baroque, Romantic	(+)	(+)	(+)	Individual	M. Medicine	045 min.	021x	7x/week	3w 0d
		(–)	(–)	(–)	**Short Stories** (from Hungary)	(+)	(+)	(+)						
**Hendricks** et al.	**1999**	(+)	(+)	(+)	Relax-Music	(+)	(+)	(+)	Group	M. Medicine	(–?–)	008x	1x/week	8w 0d
**Hsu** and **Lai**	**2004**	(+)	(+)	(–)	**Classical**, Relax-Music, Asian	(+)	(+)	(–)	Individual	M. Medicine	030 min.	014x	7x/week	2w 0d
**Kim** et al.	**2006**	(+)	(+)	(–)	Rock and Pop Music	(+)	(+)	(–)	Individual	M. Medicine	030 min.	009x	3x/week	3w 0d
**Koelsch** et al.	**2010**	(+)	(–)	(+)	**Classical**, **Jazz**, Salsa, Reggae	(+)	(+)	(–)	Group	M. Medicine	060 min.	001x	1x/week	0w 1d
**Lu** et al.	**2013**	(+)	(+)	(+)	**Drumming**, Percussion	(+)	(+)	(–)	Group	M. Medicine	060 min.	010x	2x/week	5w 0d
**Schwantes** and **McKinney**	**2010**	(+)	(+)	(+)	**Drumming**, Guitar, Piano	(–)	(–)	(–)	Group	**M. Therapy**	075 min.	004x	1x/week	4w 0d
**Silverman**	**2011**	(+)	(+)	(+)	12-bar BLUES, Blues Songwriting	(–)	(–)	(–)	Group	**M. Therapy**	045 min.	001x	1x/week	0w 1d
**Verrusio** et al.	**2014**	(+)	(+)	(–)	**Classical**, **Jazz**, Modern Music	(+)	(+)	(–)	Group	M. Medicine	060 min.	048x	2x/week	24w 0d
**Wang** et al.	**2011**	(–?–)	(–?–)	(–)	Relax-Music, Rhythmic Music	(+)	(+)	(–)	Group	M. Medicine	(–?–)	(–?–)	(–?–)	(–?–)

AUTHOR	YEAR	DEPRESSION MEASUREMENT METHODS: TEST	Experimental PRE (SD)	Experimental POST (SD)	Experimental Mean diff	Experimental Change [%]	Experimental (p)-Level	Control PRE (SD)	Control POST (SD)	Control Mean diff	Control Change [%]	Control (p)-Level	CONTROL Group(s): Alternative Treatment	Additional Notes and Comments	Effect-Size [r]
**Albornoz**	**2011**	**BDI**	18.66 (8.30)	10.58 (2.23)	[–]08.08	43.30%	p < 0.005	14.91 (4.46)	12.66 (5.28)	[–]02.25	15.09%	**p < 0.05**	—	**HAM-D** was **not** used for/in our Review	-0.249
		**HAM-D**	19.16 (5.33)	11.33 (2.53)	[–]07.83	40.87%	p < 0.005	20.00 (7.24)	16.16 (7.08)	[–]03.84	19.20%	p < 0.05			-0.414
**Ashida**	**2000**	**CSDD**	06.25 (–?–)	02.85 (–?–)	[–]03.40	54.40%	p < 0.05	(–)	(–)	(–)	—	(–)	—	**No** Control	—
**Castillo-Pérez** et al.	**2010**	**HAM-D**	(–)	(–)	(–?–)	(–?–)	p < 0.05	(–)	(–)	(–?–)	(–?–)	p > 0.05	Conductive-Behavioral PSYCHO-THERAPY	**BDI & SDS:** Data / Results were **n/a (!)** **HAM-D**: Only (p)-Level publ.	—
		**BDI**	(–)	(–)	(–?–)	(–?–)	(–?–)	(–)	(–)	(–?–)	(–?–)	(–?–)			—
		**SDS**	(–)	(–)	(–?–)	(–?–)	(–?–)	(–)	(–)	(–?–)	(–?–)	(–?–)			—
**Chan** et al.	**2009**	**GDS-30**	13.10 (5.20)	07.90 (3.50)	[–]05.20	39.69%	p < 0.005	13.40 (4.40)	15.80 (4.00)	[**+**]02.40	**-**17.91%	p = 0.007*	Rest-Period	*Significant Dep. **INCREASE**	-0.724
**Chan** et al.	**2010**	**GDS-15**	04.10 (4.00)	02.10 (3.00)	[–]02.00	48.78%	p < 0.001	01.80 (1.70)	02.00 (2.40)	[**+**]00.20	**-**11.11%	p > 0.05	Rest-Period	—	0.018
**Chan** et al.	**2012**	**GDS-15**	04.17 (3.14)	01.38 (1.84)	[–]02.79	66.91%	p < 0.05	04.23 (2.89)	04.15 (3.53)	[–]00.08	1.89%	p > 0.05	Rest-Period	—	-0.441
**Chang** et al.	**2008**	**EPDS**	12.11 (3.54)	10.27 (4.05)	[–]01.84	15.19%	p < 0.001	12.17 (3.92)	12.14 (4.60)	[–]00.03	0.25%	p > 0.05	—	—	-0.211
**Chen** et al.	**2016**	**BDI**	24.72 (10.18)	11.51 (7.78)	[–]13.21	53.44%	p < 0.01	23.90 (11.11)	20.32 (12.47)	[–]03.58	14.98%	p > 0.05	—	—	-0.390
**Choi** et al.	**2008**	**BDI**	49.30 (3.10)	25.50 (2.20)	[–]23.80	48.28%	p < 0.001	47.40 (2.80)	44.80 (3.80)	[–]02.90	5.49%	p > 0.05	—	—	-0.952
**Deshmukh** et al.	**2009**	**MADRS**	17.80 (3.61)	13.20 (3.56)	[–]04.60	25.84%	p > 0.05	17.12 (4.07)	13.68 (4.36)	[–]03.44	20.09%	p > 0.05		—	-0.060
**Erkkilä** et al.	**2011**	**MADRS**	24.60 (6.40)	14.10 (8.77)	[–]10.70	42.68%	p < 0.05	23.00 (7.60)	16.43 (9.33)	[–]06.05	28.57%	p > 0.05	—	—	-0.128
**Esfandiari** and **Mansouri**	**2014**	**BDI**	34.50 (–?–)	17.00 (–?–)	[–]17.50	50.72%	p < 0.05	32.00 (–?–)	35.00 (–?–)	[**+**]03.00	**-**9.38%	p > 0.05	—	**Two EXP One CON** Group Setting	—
		**BDI**	29.00 (–?–)	8.00 (–?–)	[–]21.00	72.41%	p < 0.05								—
**Fancourt** et al.	**2016**	**HADS-D**	08.90 (0.79)	05.48 (0.62)	[–]03.42	38.43%	p < 0.001	04.27 (1.10)	04.73 (0.87)	[**+**]00.07	**-**10.77%	p > 0.05	—	—	0.445
**Guétin, Portet** et al.	**2009**	**GDS-30**	16.70 (6.20)	08.90 (3.30)	[–]07.80	46.71%	p < 0.01	11.80 (7.40)	11.20 (6.10)	[–]00.60	5.08%	p > 0.05	Reading Session	—	-0.228
**Guétin, Soua** et al.	**2009**	**HADS-D**	06.10 (2.90)	04.90 (3.40)	[–]01.20	19.67%	p > 0.05	(–)	(–)	—	—	(–)	—	**No** Control	—
**Gupta** and Gupta	**2005**	**BDI**	08.94 (3.01)	06.24 (2.14)	[–]02.70	30.20%	p < 0.001	08.76 (3.01)	08.49 (3.59)	[–]00.27	3.08%	p > 0.05	—		-0.356
**Han** et al.	**2011**	$RMBPC_D$	20.50 (23.50)	11.70 (15.90)	[–]08.80	42.93%	p < 0.05	13.10 (21.00)	24.60 (34.70)	[**+**]11.50	**-**87.79%	p > 0.05	—	—	-0.232
		**AES**	18.20 (6.40)	19.0 (4.80)	[+]00.80	**-4.40%**	**p > 0.05**	17.10 (04.30)	16.60 (05.10)	[–]00.50	2.92%	p > 0.05			0.235
**Hanser** and **Thompson**	**1994**	**GDS-30**	17.30 (5.85)	7.70 (3.66)	[–]09.60	55.49%	p < 0.05	15.30 (5.85)	16.20 (6.13)	[**+**]00.90	**-**5.88%	p > 0.05	—	**Two EXP One CON** Group Setting	-0.644
		**GDS-30**	17.60 (7.89)	12.30 (8.65)	[–]05.30	30.11%	p < 0.05								-0.252
**Harmat** et al.	**2008**	**BDI**	5.40 (3.767)	2.66 (2.06)	[–]02.74	50.74%	p < 0.05	n/a	n/a	(–)	(–)	p > 0.05	—	—	—
		**BDI**	5.70 (3.564)	5.13 (3.33)	[–]00.57	10.00%	**p > 0.05**								
**Hendricks** et al.	**1999**	**BDI**	39.00 (–?–)	1.34 (–?–)	[–]37.66	96.56%	p < 0.05	32.30 (–?–)	17.00 (–?–)	[–]15.30	47.37%	**p < 0.05**	Cognitive-Behavioral Activities	CON Results far from EXP Group	—
**Hsu** and **Lai**	**2004**	**SDS**	81.34 (6.39)	051.39 (6.21)	[–]29.95	36.82%	p < 0.001	80.60 (5.34)	62.17 (7.07)	[–]18.43	22.87%	p > 0.05	Rest-Period	—	-0.629
**Kim** et al.	**2006**	**SDS**	46.11 (8.67)	42.17 (8.44)	[–]03.94	8.54%	p < 0.01	46.44 (8.29)	47.44 (9.12)	[**+**]00.94	**-**2.15%	p > 0.05	—	—	-0.287
**Koelsch** et al.	**2010**	$POMS_D$	06.57 (8.01)	03.51 (5.91)	[–]03.06	46.58%	p < 0.001	08.25 (10.37)	09.30 (9.68)	[**+**]01.05	**-**12.73%	p > 0.05	—	—	-0.340
**Lu** et al.	**2013**	**CDSS**	04.23 (4.78)	0.89 (1.62)	[–]03.34	78.96%	p < 0.005	3.38 (4.65)	03.33 (4.25)	[–]00.05	1.48%	p > 0.05	—	—	-0.355
**Schwantes** and **McKinney**	**2010**	**CES-D**	21.60 (3.22)	15.60 (2.66)	[–]06.00	27.78%	p < 0.05	(–)	(–)	(–)	—	(–)	—	—	—
**Silverman**	**2011**	**BDI**	n/a	18.79 (9.14)	(–?–)	(–?–)	**p > 0.05**	n/a	20.28 (9.53)	(–?–)	(–?–)	p > 0.05	Verbal Therapy	Only **Post**-Results shared	-0.080
**Verrusio** et al.	**2014**	**GDS-15**	08.50 (2.20)	05.50 (1.00)	[–]03.00	35.29%	p < 0.01	08.40 (1.80)	08.00 (2.50)	[–]00.40	4.76%	p > 0.05	Medication	—	-0.549
**Wang** et al.	**2011**	**SDS**	45.45 (5.23)	40.21 (5.10)	[–]05.24	11.53%	p < 0.001	46.01 (5.34)	46.19 (5.07)	[**+**]00.18	**-**0.39%	p > 0.05	—	—	-0.507
