# Supplementary material for: Reviewing the Effectiveness of Music Interventions in Treating Depression
Source: Front Psychol. 2017 Jul 7;8:1109. doi: 10.3389/fpsyg.2017.01109 (PMC5500733; doi:10.3389/fpsyg.2017.01109)
Supplement: Supplementary file 3 [file DataSheet3.pdf]

Title / Author / Year	Questionnaires	Results
**Albornoz**<br>(2011)<br><br>The effects of group improvisational music therapy on depression in adolescents and adults with substance abuse: a randomized controlled trial	**BDI**<br>Beck's Depression Inventory<br><br>**HAM-D** (=) **HRSD**<br>Hamilton Rating Scale for Depression	**BDI**-Score:<br>• **Experimental** (=) Group music-therapy [mean (SD)]<br>• PRE (D0) 18.66 (SD 8.30)<br>• POST (W36) 10.58 (SD 2.23)<br>• **Control** group (=) Standard treatment [mean (SD)]<br>• PRE (D0) 14.91 (SD 4.46)<br>• POST (W36) 12.66 (SD 5.28)<br>⇨ **Significant** decrease in the score for depression within the experimental (=) music group (($p=0.002$) ($p<0.005$))<br>⇨ **Significant** decrease in the score for depression within the control group (($p=0.04$) ($p<0.05$))<br>⇨ **No** significant difference between the 2 groups at any time ($p>0.05$)<br><br>**HAM-D**-Score:<br>• **Experimental** (=) Group music-therapy [mean (SD)]<br>• PRE (D0) 19.16 (SD 5.33)<br>• POST (W36) 11.33 (SD 2.53)<br>• **Control** group (=) Standard treatment [mean (SD)]<br>• PRE (D0) 20.00 (SD 7.24)<br>• POST (W36) 16.16 (SD 7.08)<br>⇨ **Significant** decrease in the score for depression in the experimental (=) music group (means differed by 7.83 points (($p=0.002$) ($p<0.005$))<br>⇨ **Significant** decrease in the score for depression in the control group (means differed by 3.84 points (($p=0.01$) ($p<0.05$))<br>⇨ **Significant** difference between the 2 groups on post-test HRSD scores ($p>0.05$) (*experimental group had significantly lower post-test score than the control*)

Title / Author / Year	Questionnaires	Results
**Ashida**<br>(2000)<br><br>The Effect of Reminiscence Music Therapy Sessions on Changes in Depressive Symptoms in Elderly Persons with Dementia	**CSDD**<br>Cornell Scale for Depression in Dementia	**CSDD**-Score:<br>• **Experimental** (=) Music group [mean (SD)]<br>• PRE (D0) 6.25 (SD n/a)<br>• POST (W3) 2.85 (SD n/a)<br>• **Control** group [mean (SD)]<br>• Not used<br>⇨ **Significant** decrease in the score for depression within the experimental (=) music group ($p<0.05$)<br>⇨ **Significant** differences of score in the experimental (=) music group between<br>• **Pre**-Test and **Post**-Test-**2**<br>• **Post**-Test **1** and **Post**-Test-**2**<br>⇨ **No** significant differences of score in the experimental (=) music group between<br>• **Pre**-Test and **Post**-Test-**1**

Title / Author / Year	Questionnaires	Results
**Castillo-Pérez** et al. (2010)<br><br>Effects of music therapy on depression compared with psychotherapy	**BDI**<br>Beck's Depression Inventory<br><br>**HAM-D** (=) **HRSD**<br>Hamilton Rating Scale for **Depression**<br>⇨ Final evaluation only!<br><br>**SDS**<br>(Zung's) Self-Rating Depression Scale (Chinese version)<br>⇨ Initial selection only!	**BDI**-Score:<br>• **Experimental** (=) Music group [mean (SD)]<br>• PRE (D0) n/a (SD n/a)<br>• POST (W8) n/a (SD n/a)<br>• **Control** group (=) Psychotherapy [mean (SD)]<br>• PRE (D0) n/a (SD n/a)<br>• POST (W8) n/a (SD n/a)<br>⇨ **Significant** decrease in the score for depression within the experimental (=) music group ((p=0.0356) (p<0.05) was confirmed by the authors)<br>⇨ **No** significant decrease in the score for depression within the control group (p>0.05)<br><br>**SDS**-Score:<br>• **Experimental** (=) Music group [mean (SD)]<br>• PRE (D0) n/a (SD n/a)<br>• POST (W8) n/a (SD n/a)<br>• **Control** group (=) Psychotherapy [mean (SD)]<br>• PRE (D0) n/a (SD n/a)<br>• POST (W8) n/a (SD n/a)<br><br>**HAM-D**-Score (final evaluation only):<br>• **Experimental** (=) Music group [mean (SD)]<br>• PRE (D0) n/a (SD n/a)<br>• POST (W8) n/a (SD n/a)<br>• **Control** group (=) Psychotherapy [mean (SD)]<br>• PRE (D0) n/a (SD n/a)<br>• POST (W8) n/a (SD n/a)

Title / Author / Year	Questionnaires	Results
**Chan** et al. (2009)<br><br>Effect of music on depression levels and physiological responses in community-based older adults	**GDS-30**<br>Geriatric Depression Scale (30 items version) (Chinese translation)	**GDS(-30)**-Score:<br>• **Experimental** (=) Music group [mean (SD)]<br>• PRE (W0) 13.1 (SD 5.2)<br>• POST (W4) 07.9 (SD 3.5)<br>• **Control** (=) Resting period group [mean (SD)]<br>• PRE (W0) 13.4 (SD 4.4)<br>• POST (W4) 15.8 (SD 4.0)<br>⇨ **Significant** decrease in the score for depression within the experimental (=) music group ((p=0.001) (p<0.005))<br>⇨ **No** significant decrease but instead a **significant <u>in</u>crease** in the depression score within the control (=) resting period group ((p=0.007) (p<0.01))<br>⇨ **Significant** difference in the score for depression between the experimental (=) music and the control (=) resting period group (p<0.001)

Title / Author / Year	Questionnaires	Results
**Chan** et al. (2010)<br><br>Effects of music on depression and sleep quality in elderly people: A randomised controlled trial	**GDS-15**<br>Geriatric Depression Scale (15 items version) (Chinese translation)<br><br>**PSQI**<br>Pittsburgh Sleep Quality Index	**GDS(-15)**-Score:<br>• **Experimental** (=) Music listening [mean (SD)]<br>• PRE (W1) 4.1 (SD 4.0)<br>• POST (W4) 2.1 (SD 3.0)<br>• **Control** group (=) Rest period [mean (SD)]<br>• PRE (W1) 1.8 (SD 1,7)<br>• POST (W4) 2.0 (SD 2.4)<br>⇨ **Significant** decrease in the score for depression within the experimental (=) music group (p<0.001)<br>⇨ **No** significant decrease in the score for depression within the control group ((p=0.791) (p>0.05))<br><br>**PSQI**-Score (lower scores (=) healthier sleep quality):<br>• **Experimental** (=) Music listening [mean (SD)]<br>• PRE (W1) 7.6 (SD 4.0)<br>• POST (W4) 5.1 (SD 2.6)<br>• **Control** group (=) Rest period [mean (SD)]<br>• PRE (W1) 6.1 (SD 3.7)<br>• POST (W4) 6.0 (SD 3.6)<br>⇨ **Significant** decrease in the score for sleep quality within the experimental (=) music group (p<0.001)<br>⇨ **No** significant decrease in the score for sleep quality within the control group ((p=0.252) (p>0.05))
**Chan** et al. (2012)<br><br>Effects of music on depression in older people: a randomised controlled trial	**GDS-15**<br>Geriatric Depression Scale (15 items version)	**GDS(-15)**-Score:<br>• **Experimental** (=) Music group [mean (SD)]<br>• PRE (W1) 4.17 (SD 3.14)<br>• POST (W8) 1.38 (SD 1.84)<br>• **Control** (=) Resting period group [mean (SD)]<br>• PRE (W1) 4.23 (SD 2.89)<br>• POST (W8) 4.15 (SD 3.53)<br>⇨ **Significant** decrease in the score for depression within the experimental (=) music group ((p=0.016) (p<0.05))<br>⇨ **Significant** differences in the depression scores between the experimental (=) music and the control group were found at week 8 ((p=0.006) (p<0.01))<br>⇨ **No** significant decrease in the score for depression within the control group ((p=0.677) (p>0.05))

Title / Author / Year	Questionnaires	Results
**Chang** et al.<br>(2008)<br><br>Effects of music therapy on psychological health of women during pregnancy	**EPDS**<br>Edinburgh Postnatal Depression Scale<br><br>**PSS**<br>Perceived Stress Scale<br><br>**STAI** (**S**-STAI)<br>State-Trait Anxiety Inventory<br>• **State** anxiety sub-scale	**EPDS**-Score:<br>• **Experimental** (=) Music therapy [mean (SD)]<br>• PRE (D0) 12.11 (SD 3.54)<br>• POST (W2) 10.27 (SD 4.05)<br>• **Control** (=) General prenatal care group [mean (SD)]:<br>• PRE (D0) 12.17 (SD 3.92)<br>• POST (W2) 12.14 (SD 4.60)<br>⇨ **Significant** decrease in the score for depression within the experimental (=) music group ($p<0.001$)<br>⇨ **No** significant decrease in the score for depression within the control group ($p>0.05$)<br><br>**PSS**-Score:<br>• **Experimental** (=) Music drumming group [mean (SD)]<br>• PRE (D0) 17.44 (SD 4.56)<br>• POST (W2) 15.29 (SD 5.22)<br>• **Control** (=) Non-music activities [mean (SD)]<br>• PRE (D0) 16.71 (SD 4.31)<br>• POST (W2) 15.79 (SD 5.99)<br>⇨ **Significant** decrease in the PSS score for stress within the experimental (=) music group ($p<0.001$)<br>⇨ **Significant** decrease in the PSS score for stress within the control group ($p<0.05$)<br><br>**STAI**-Score (**S**-STAI) **State** anxiety sub scale:<br>• **Experimental** (=) Music group [mean (SD)]<br>• PRE (D0) **State** 37.92 (SD 09.79)<br>• POST (W2) **State** 35.79 (SD 10.86)<br>• **Control** group (=) No intervention [mean (SD)]<br>• PRE (D0) **State** 37.08 (SD 10.04)<br>• POST (W2) **State** 37.79 (SD 12.11)<br>⇨ **Significant** decrease in the STAI score for state anxiety within the experimental (=) music group ($p<0.05$)<br>⇨ **No** significant decrease in the STAI score for state anxiety within the control group ($p>0.05$)

Title / Author / Year	Questionnaires	Results
**Chen** et al.<br>(2016)<br><br>Randomized Trial of Group Music Therapy With Chinese Prisoners: Impact on Anxiety, Depression, and Self-Esteem<br><br>*Based on and includes data from: “Chen et al. (2014): Group music therapy for prisoners: Protocol for a randomised controlled trial”*	**BDI**<br>Beck’s Depression Inventory<br><br>**STAI**<br>State-Trait Anxiety Inventory<br>• **State** anxiety sub-scale<br>• **Trait** anxiety sub-scale<br><br>**RSI (=) SEI**<br>Rosenberg Self-Esteem Inventory<br><br>**TSBI**<br>Texas Social Behaviour Inventory (Chinese version)	**BDI**-Score:<br>• **Experimental** (=) Music group [mean (SD)]<br>• PRE (W0) 24.72 (SD 10.18)<br>• POST (W10) 11.51 (SD 07.78)<br>• **Control** group (=) Usual care [mean (SD)]<br>• PRE (W0) 23.90 (SD 11.11)<br>• POST (W10) 20.32 (SD 12.47)<br>⇨ **Significant** decrease in the score on depression within the experimental (=) music intervention group ($p<0.01$)<br>⇨ **No** significant decrease in the depression score within the control (=) usual care group ($p>0.05$)<br><br>**STAI**-Score - **State** anxiety / **Trait** anxiety:<br>• **Experimental** (=) Music group [mean (SD)]<br>• PRE (D0) **State** 48.52 (SD 9.67) // **Trait** 48.70 (SD 08.98)<br>• POST (Dn/a) **State** 40,53 (SD 8.74) // **Trait** 40.58 (SD 08.47)<br>• **Control** group (=) Usual care [mean (SD)]<br>• PRE (D0) **State** 48.03 (SD 9.95) // **Trait** 48.52 (SD 10.12)<br>• POST (Dn/a) **State** 48.58 (SD 9.86) // **Trait** 49.09 (SD 08.17)<br>⇨ **Significant** decrease of score for state and trait anxiety within the experimental (=) music intervention group (($p<0.001$) for state anxiety; ($p<0.001$) for trait anxiety)<br>⇨ **Significant** differences in outcome scores for state and trait anxiety at post-treatment condition (($p<0.02$) for state anxiety; ($p<0.05$) for trait anxiety)<br>⇨ **No** significant decrease but increase of score for state and trait anxiety within the control (=) usual care group (($p>0.05$) for state anxiety; ($p>0.05$) for trait anxiety)

Title / Author / Year	Questionnaires	Results
**Chen** et al. (2016)<br><br>… ***continued* from above:**<br><br>Randomized Trial of Group Music Therapy With Chinese Prisoners: Impact on Anxiety, Depression, and Self-Esteem	**RSI (=) SEI**<br>Rosenberg Self-Esteem Inventory	**RSI (=) SEI**-Score (higher = more self-esteem):<br>• **Experimental** (=) Music group [mean (SD)]<br>• PRE (W0) 25.92 (SD 4.11)<br>• POST (W10) 29.27 (SD 4.25)<br>• **Control** group (=) Usual care [mean (SD)]<br>• PRE (W0) 26.04 (SD 3.65)<br>• POST (W10) 27.01 (SD 4.60)<br>⇨ **Significantly** higher RSI-scores for self-esteem in the experimental (=) music group compared to the control (=) usual care group at post-treatment condition (p<0.001)<br>⇨ **Significant** decrease in the RSI-score within the experimental (=) music group (p<0.001)<br>⇨ **No** significant decrease in the RSI-score within the control (=) usual care group (p>0.05)
	**TSBI**<br>Texas Social Behaviour Inventory (Chinese version)	**TSBI**-Score (higher (=) better (more self-esteem)):<br>• **Experimental** (=) Music group [mean (SD)]<br>• PRE (W0) 97.30 (SD 15.34)<br>• POST (W10) 104.35 (SD 13.62)<br>• **Control** group (=) Usual care [mean (SD)]<br>• PRE (W0) 97.56 (SD 15.22)<br>• POST (W10) 96.81 (SD 16.00)<br>⇨ **Significantly** higher TSBI-scores for self-esteem in the experimental (=) music group compared to the control (=) usual care group at post-treatment condition ((p=0.001) (p<0.005))<br>⇨ **Significant** increase in the TSBI-score for self-esteem within the experimental (=) music group (p<0.05)<br>⇨ **No** significant increase in the TSBI-scores for self-esteem within the control (=) usual care group (p>0.05)

Title / Author / Year	Questionnaires	Results
**Choi** et al.<br>(2008)<br><br>Effects of Group Music Intervention on Depression, Anxiety, and Relationships in Psychiatric Patients: A Pilot Study	**BDI**<br>Beck's Depression Inventory<br><br>**STAI**<br>State-Trait Anxiety Inventory<br><br>**RCS**<br>Relationship Change Scale<br>• (W**0**) = Baseline<br>• (W**p**) = Post-intervention	**BDI**-Score:<br>• **Experimental** (=) Music group [mean (SD)]<br>• PRE (W0) 49.3 (SD 3.1)<br>• POST (Wp) 25.5 (SD 2.2)<br>• **Control** group (=) Usual care [mean (SD)]<br>• PRE (W0) 47.7 (SD 2.8)<br>• POST (Wp) 44.8 (SD 3.8)<br>⇨ **Significant** decrease in the BDI score for depression within the experimental (=) music intervention group (p<0.001)<br>⇨ **No** significant decrease in the BDI score for depression within the control group (p>0.05)<br><br>**STAI**-Score - **State** anxiety / **Trait** anxiety (W**0** = Baseline; W**p** = Post interven.)<br>• **Experimental** (=) Music group [mean (SD)]<br>• PRE (W0) **State** 36.5 (SD 0.8) // **Trait** 35.9 (SD 1.0)<br>• POST (Wp) **State** 22.8 (SD 1.7) // **Trait** 23.0 (SD 1.6)<br>• **Control** group (=) Usual care [mean (SD)]<br>• PRE (W0) **State** 36.2 (SD 1.2) // **Trait** 36.4 (SD 1.6)<br>• POST (Wp) **State** 32.5 (SD 1.7) // **Trait** 34.2 (SD 1.1)<br>⇨ **Significant** decrease in the score for state and trait anxiety within the experimental (=) music intervention group ((p<0.001) for state anxiety; (p<0.001) for trait anxiety)<br>⇨ **No** significant decrease in the score for state or trait anxiety within the control group ((p>0.05) for state anxiety; (p>0.05) for trait anxiety)<br><br>**RCS**-Score:<br>• **Experimental** (=) Music group [mean (SD)]<br>• PRE (W0) 72.4 (SD 1.2)<br>• POST (Wp) 45.8 (SD 2.9)<br>• **Control** group (=) Usual care [mean (SD)]<br>• PRE (W0) 72.5 (SD 2.3)<br>• POST (Wp) 66.7 (SD 2.0)<br>⇨ **Significant** decrease (i.e. improvement) in the relationship score within the experimental (=) music group (p<0.001)<br>⇨ **No** significant decrease (i.e. improvement) in the relationship score within the control group (p>0.05)

Title / Author / Year	Questionnaires	Results
**Deshmukh** et al. (2009)<br><br>Effect of Indian classical music on quality of sleep in depressed patients: A randomized controlled trial	**MADRS**<br>Montgomery–Åsberg Depression Rating Scale<br><br>**PSQI**<br>Pittsburgh Sleep Quality Index	**MADRS**-Score:<br>• **Experimental** (=) Music group [mean (SD)]<br>• PRE (D0) 17.80 (SD 3.61)<br>• POST (D45) 13.20 (SD 3.56)<br>• **Control** group (=) No music intervention [mean (SD)]<br>• PRE (D0) 17.12 (SD 4.07)<br>• POST (D45) 13.68 (SD 4.36)<br>⇨ **No** significant decrease in the score on depression within the experimental (=) music group (p>0.05)<br>⇨ **No** significant decrease in the score on depression within the control group (p>0.05)<br><br>**PSQI**-Score (lower scores (=) healthier sleep quality):<br>• **Experimental** (=) Music group [mean (SD)]<br>• PRE (D0) 12.20 (SD 2.02)<br>• POST (D45) 08.36 (SD 2.69)<br>• **Control** group (=) No music intervention [mean (SD)]<br>• PRE (D0) 12.04 (SD 2.07)<br>• POST (D45) 09.64 (SD 2.06)<br>⇨ **No** significant decrease in the score for sleep quality within the experimental (=) music group (p>0.05)<br>⇨ **No** significant decrease in the score for sleep quality within the control group (p>0.05)<br>⇨ **Significant** difference of PSQI scores between the experimental (=) music and the control group (p>0.05)

Title / Author / Year	Questionnaires	Results
**Erkkilä** et al. (2011)<br><br>Individual music therapy for depression: randomised controlled trial	**MADRS**<br>Montgomery–Åsberg Depression Rating Scale<br><br>**HAD-A (=) HADSA**<br>Hospital Anxiety and Depression Scale<br>• **A**nxiety subscale<br><br>**HRQOL**<br>Health-Related Quality of Life Survey (SF-36) (short version with 36 items)	**MADRS**-Score:<br>• **Experimental** (=) Music therapy group [mean (SD)]<br>• PRE (W0) 24.60 (SD 6.40)<br>• POST (W12) 14.10 (SD 8.77)<br>• **Control** group (=) Standard care [mean (SD)]<br>• PRE (W0) 23.00 (SD 7.60)<br>• POST (W12) 16.43 (SD 9.33)<br>⇨ **Significant** decrease in the score for depression within the experimental (=) music group (p<0.05)<br>⇨ **No** significant decrease in the score for depression in the control group (p>0.05)<br><br>**HAD**-Test (**Anxiety** sub-scale):<br>• **Experimental** (=) Music group [mean (SD)]<br>• PRE (W0) 11.20 (SD 3.50)<br>• POST (W12) 07.37 (SD 3.99)<br>• **Control** group (=) Standard care [mean (SD)]<br>• PRE (W0) 10.30 (SD 3.90)<br>• POST (W12) 08.00 (SD 4.11)<br>⇨ **Significant** decrease in the score for anxiety within the experimental (=) music group (p<0.05)<br>⇨ **No** significant decrease in the score on anxiety in the control group (p>0.05)<br><br>**HRQOL**-Score:<br>• **Experimental** (=) Music therapy group [mean (SD)]<br>• PRE (W0) 50.50 (SD 15.30)<br>• POST (W12) 66.70 (SD 20.10)<br>• **Control** group (=) Standard care [mean (SD)]<br>• PRE (W0) 52.60 (SD 13.90)<br>• POST (W12) 62.59 (SD 18.20)<br>⇨ **No** significant decrease of the Health-Related Quality of Life Survey score in the experimental (=) music group ((p=0.20) (p>0.05))<br>⇨ **No** significant decrease of the Health-Related Quality of Life Survey score in the control group (p>0.05)

Title / Author / Year	Questionnaires	Results
**Esfandiari** and **Mansouri** (2014)<br><br>The effect of listening to light and heavy music on reducing the symptoms of depression among female students	**BDI**<br>Beck's Depression Inventory<br><br>**DSM-IV**<br>Diagnostic and Statistical Manual of Mental disorder (4th edition)	**BDI**-Score:<br>• **Experimental-1** (=) Light Music group [mean (SD)]<br>• PRE (D0) 34.50 (SD n/a)<br>• POST (W8) 17.00 (SD n/a)<br>• **Experimental-2** (=) Heavy Music group [mean (SD)]<br>• PRE (D0) 29.00 (SD n/a)<br>• POST (W8) 08.00 (SD n/a)<br>• **Control** group (=) No music intervention [mean (SD)]<br>• PRE (D0) 32.00 (SD n/a)<br>• POST (W8) 35.00 (SD n/a)<br>⇨ **Significant** decrease in the depression score within the "**light**" music" (=) experimental group **no. 1** ($p<0.05$)<br>⇨ **Significant** decrease in the score on depression in the "**heavy**" music (=) experimental group **no. 2** ($p<0.05$)<br>⇨ **No** decrease in the score on depression in the control group ($p>0.05$)

Title / Author / Year	Questionnaires	Results
**Fancourt et al.**<br>(2016)<br><br>Effects of Group Drumming Interventions on Anxiety, Depression, Social Resilience and Inflammatory Immune Response among Mental Health Service Users	**HAD (=) HADS(A/D)**<br>Hospital Anxiety and Depression Scale<br>HADS**A** for **A**nxiety<br>HADS**D** for **D**epression<br><br>**PSS**<br>Perceived Stress Scale<br><br>**WEMWBS**<br>Warwick-Edinburgh Mental Wellbeing Scale (wellbeing)<br><br>**CDRISC**<br>Connor-Davidson Resilience Scale (for measuring social function)	**HAD**-Test (Depression (**DEP**) // Anxiety (**ANX**) sub-scale):<br>• **Experimental** (=) Music drumming group [mean (SD)]<br>• PRE (D0) **ANX** 11.03 (SD 0.83)<br>• POST (W10) **ANX** 08.83 (SD 0.70)<br>• PRE (D0) **DEP** 08.90 (SD 0.79)<br>• POST (W10) **DEP** 05.48 (SD 0.62)<br>• **Control** (=) Non-music activities [mean (SD)]<br>• PRE (D0) **ANX** 09.93 (SD 1.16)<br>• POST (W10) **ANX** 09.60 (SD 0.97)<br>• PRE (D0) **DEP** 04.27 (SD 1.10)<br>• POST (W10) **DEP** 04.73 (SD 0.87)<br>⇨ **Significant** decrease of score at week 10 for anxiety and depression HAD sub-scales within the experimental (=) music group (($p<0.05$) for the anxiety sub-scale; ($p<0.001$) for the depression sub-scale)<br>⇨ **No** significant decrease of score at week 10 for anxiety and depression HAD sub-scales within the control (=) non-music activities group (($p>0.05$) for the anxiety sub-scale); ($p>0.05$) for the depression sub-scale)<br><br>**PSS**-Score:<br>• **Experimental** (=) Music drumming group [mean (SD)]<br>• PRE (D0) 23.17 (SD 1.28)<br>• POST (W10) 19.52 (SD 1.12)<br>• **Control** (=) Non-music activities [mean (SD)]<br>• PRE (D0) 21.87 (SD 1.78)<br>• POST (W10) 16.00 (SD 1.55)<br>⇨ **Significant** decrease in the PSS score at week 10 within the experimental (=) music group ($p<0.05$)<br>⇨ **No** significant decrease in the PSS score at week 10 within the control (=) non-music activities group ($p>0.05$)

Title / Author / Year	Questionnaires	Results
**Fancourt et al.** (2016)<br><br>***… continued from above:***<br><br>Effects of Group Drumming Interventions on Anxiety, Depression, Social Resilience and Inflammatory Immune Response among Mental Health Service Users	**WEMWBS** Warwick-Edinburgh Mental Wellbeing Scale (wellbeing)<br><br>**CDRISC** Connor-Davidson Resilience Scale (for measuring social function)	**WEMWBS**-Score:<br>• **Experimental** (=) Music drumming group [mean (SD)]<br>• PRE (D0) 39.61 (SD 1.91)<br>• POST (W10) 45.75 (SD 1.80)<br>• **Control** (=) Non-music activities [mean (SD)]<br>• PRE (D0) 44.67 (SD 2.61)<br>• POST (W10) 47.00 (SD 2.46)<br>⇨ **Significant** decrease in the WEMWBS score at week 10 within the experimental (=) music group (p<0.05)<br>⇨ **No** significant decrease in the WEMWBS score at week 10 within the control (=) non-music activities group (p>0.05)<br><br>**CDRISC**-Score:<br>• **Experimental** (=) Music drumming group [mean (SD)]<br>• PRE (D0) 46.93 (SD 3.47)<br>• POST (W10) 57.52 (SD 3.16)<br>• **Control** (=) Non-music activities [mean (SD)]<br>• PRE (D0) 57.85 (SD 4.83)<br>• POST (W10) 59.07 (SD 4.39)<br>⇨ **Significant** decrease in the CDRISC score at week 10 within the experimental (=) music group (p<0.05)<br>⇨ **No** significant decrease in the CDRISC score at week 10 within the control (=) no music intervention group (p>0.05)

Title / Author / Year	Questionnaires	Results
**Guétin, Portet** et al. (2009)<br><br>Effect of Music Therapy on Anxiety and Depression in Patients with Alzheimer's Type Dementia: Randomised, Controlled Study	**GDS-30**<br>30-item Geriatric Depression Scale<br><br>**HAM-A**<br>Hamilton **Anxiety** Rating Scale<br><br>**MMSE**<br>Mini–Mental State Examination (detects cognition changes)<br>⇨ Additional analysis	**GDS(-30)**-Score:<br>• **Experimental** group (Music therapy) [mean (SD)]<br>• PRE (D0) 16.7 (SD 6.2)<br>• POST (W16) 08.9 (SD 3.3)<br>• **Control** group [mean (SD)]<br>• PRE (D0) 11.8 (SD 7.4)<br>• POST (W16) 11.2 (SD 6.1)<br>⇨ **Significant** decrease in the depression score at week 16 within the experimental (=) music group (p<0.01)<br>⇨ **No** significant decrease in the depression score at week 16 within the control (=) reading group (p>0.05)<br><br>**HAM-A**-/ (=) **HAS**-Score:<br>• **Experimental** group (Music therapy) [mean (SD)]<br>• PRE (D0) 22.0 (SD 5.3)<br>• POST (W16) 08.4 (SD 3.7)<br>• **Control** group [mean (SD)]<br>• PRE (D0) 21.1 (SD 5.6)<br>• POST (W16) 20.8 (SD 6.2)<br>⇨ **Significant** decrease in the anxiety score at week 16 within the experimental (=) music group (p<0.01)<br>⇨ **No** significant decrease in the anxiety score at week 16 within the control (=) reading group (p>0.05)<br><br>**MMSE**-Score:<br>• **Experimental** group (Music therapy) [mean (SD)]<br>• PRE (D0) 19.8 (SD 4.4)<br>• POST (W16) 19.6 (SD 4.4)<br>• **Control** group [mean (SD)]<br>• PRE (D0) 20.7 (SD 3.4)<br>• POST (W16) 19.8 (SD 3.3)<br>⇨ **No** significant differences in the Mini–Mental State Examination scores between the 2 groups at any time (p>0.05)

Title / Author / Year	Questionnaires	Results
**Guétin, Soua** et al. (2009)<br><br>The effect of music therapy on mood and anxiety-depression: An observational study in institutionalised patients with traumatic brain injury	**HAD (=) HADS(A/D)**<br>Hospital Anxiety and Depression Scale<br>• HADS**A** for **A**nxiety<br>• HADS**D** for **D**epression<br><br>**Face scale**<br>⇨ Mood measuring (patient-scored)<br><br>*Note:*<br>*We counted this article as significant, although final results were not significant for depression score decrease at week twenty.*<br>*This was due to the overall results [HADS-D] test scores for weeks five, ten and fifteen that were all significant. Only week twenty did not follow this trend of improvement. It is also important to mention that every one of the additional tests (used to measure changes in anxiety and mood) showed significant improvements for the experimental group after they received music therapy.*	**HAD**-Test (Depression (**DEP**) // Anxiety (**ANX**) sub-scale):<br>• **Experimental** (=) Music group [mean (SD)]<br>• PRE (W01) **DEP** 6.1 (SD 2.9) // **ANX** 9.8 (SD 5.0)<br>• Late (W15) **DEP** 4.6 (SD 2.9) // **ANX** 6.5 (SD 2.0)<br>• POST (W20) **DEP** 4.9 (SD 3.4) // **ANX** 6.0 (SD 3.4)<br>• **Control** group [mean (SD)]<br>• *No control used*<br>⇨ **Significant** decrease in the score at week **15** for depression and anxiety HAD sub-scales within the experimental (=) music intervention group ((p<0.05) for the depression; (p<0.05) for the anxiety sub-scale)<br>⇨ **No** significant decrease of score for the experimental (=) music group for depression HAD sub-scale at week **20** (p>0.05) (*counted as **significant***)<br>⇨ **Significant** decrease of score at week **20** for the anxiety HAD sub-scale within the experimental (=) music intervention group (p<0.05)<br>⇨ **Significant** decrease in the HAD score within the experimental (=) music intervention group at: W**01-15**; W**15**, W**01-20**; W**20**;<br><br>**Face(-Scale)**-Scores (**Mood** measuring):<br>• **Experimental** (=) Music group [mean (SD)]<br>• "*Week-01*" (W01) Before music therapy 4.6 (SD 3.2)<br>• "*Week-01*" (W01) After music therapy 2.6 (SD 2.0)<br>• "*Week-05*" (W05) Before music therapy 3.5 (SD 3.0)<br>• "*Week-05*" (W05) After music therapy 2.0 (SD 1.8)<br>• "*Week-10*" (W10) Before music therapy 3.1 (SD 2.7)<br>• "*Week-10*" (W10) After music therapy 1.7 (SD 2.1)<br>• "*Week-15*" (W15) Before music therapy 3.2 (SD 2.7)<br>• "*Week-15* (W15) After music therapy 1.7 (SD 2.1)<br>• "*Week-20*" (W20) Before music therapy 2.8 (SD 2.0)<br>• "*Week-20*" (W20) After music therapy 1.1 (SD 1.0)<br>⇨ **Significant** decrease in the mood score for the experimental (=) music group at week **01** (before/after music intervention ((p=0.008) (p<0.01))<br>⇨ **Significant** decrease in the mood score for the experimental (=) music group at weeks **05** (before/after music intervention ((p=0.03) (p<0.05))<br>⇨ **Significant** decrease in the mood score for the experimental (=) music group at week **10** (before/after music intervention (p<0.05)<br>⇨ **Significant** decrease in the mood score for the experimental (=) music group at week **15** (before/after music intervention ((p=0.01) (p<0.05))<br>⇨ **Significant** decrease in the mood score for the experimental (=) music group at week **20** (before/after music intervention ((p=0.008) (p<0.01))

Title / Author / Year	Questionnaires	Results
**Gupta** and **Gupta** (2005)<br><br>Psychophysiological responsivity to Indian instrumental music	**BDI**<br>Beck's Depression Inventory<br><br>**STAI**<br>State-Trait Anxiety Inventory<br>• **State** anxiety sub-scale<br>• **Trait** anxiety sub-scale<br><br>**FFAI**<br>Four Factor Anxiety Inventory<br>• **Somatic** component<br>• Cognitive component<br>• Behavioral component<br>• **Affective** component	**BDI**-Score:<br>• **Experimental** (=) Music group [mean (SD)]<br>• PRE (D0) 8.94 (SD 3.01)<br>• POST (D20) 6.24 (SD 2.14)<br>• **Control** group (=) No intervention [mean (SD)]<br>• PRE (D0) 8.76 (SD 3.01)<br>• POST (D20) 8.49 (SD 3.59)<br>⇨ **Significant** decrease in the score on depression within the experimental (=) music intervention group (p<0.001)<br>⇨ **Significant** decrease in the depression score at post-treatment condition (p<0.01) for the experimental (=) music intervention compared to the control group<br>⇨ **No** significant decrease in the depression score within the control group (p>0.05)<br><br>**STAI**-Score - **State** anxiety // **Trait** anxiety:<br>• **Experimental** (=) Music group [mean (SD)]<br>• PRE (D0) **State** 48.74 (SD 9.42)<br>• POST (D20) **State** 43.86 (SD 9.98)<br>• PRE (D0) **Trait** 47.93 (SD 8.56)<br>• POST (D20) **Trait** 43.02 (SD 7.38)<br>• **Control** group (=) No intervention [mean (SD)]<br>• PRE (D0) **State** 48.81 (SD 9.39)<br>• POST (D20) **State** 49.20 (SD 9.17)<br>• PRE (D0) **Trait** 47.43 (SD 8.75)<br>• POST (D20) **Trait** 46.98 (SD 8.21)<br>⇨ **Significant** decrease of score for state and trait anxiety within the experimental (=) music intervention group ((p<0.05) for state anxiety; (p<0.01) for trait anxiety)<br>⇨ **Significant** decrease of score for state and trait anxiety at post-treatment condition for the experimental (=) music intervention compared to the control group ((p<0.02) for state anxiety; (p<0.05) for trait anxiety)<br>⇨ **No** significant decrease of score for state and trait anxiety within the control group ((p>0.05) for state anxiety; (p>0.05) for trait anxiety)

Title / Author / Year	Questionnaires	Results
**Gupta** and **Gupta** (2005)<br><br>*… __continued__ from above:*<br><br>Psychophysiological responsivity to Indian instrumental music	**FFAI**<br>Four Factor Anxiety Inventory<br>• **Somatic** component<br>• Cognitive component<br>• Behavioral component<br>• **Affective** component	**FFAI** (Somatic (**SOM**); Affective (**AFF**); (**COG**) Cognitive; (**BEH**) Behavioral):<br>• **Experimental** (=) Music group [mean (SD)]<br>• PRE (D0) **SOM** 30.65 (SD 8.78)<br>• POST (D20) **SOM** 23.14 (SD 8.05)<br>• PRE (D0) **COG** 31.47 (SD 9.04)<br>• POST (D20) **COG** 25.01 (SD 8.49)<br>• PRE (D0) **BEH** 29.24 (SD 9.25)<br>• POST (D20) **BEH** 23.03 (SD 8.27)<br>• PRE (D0) **AFF** 28.76 (SD 8.69)<br>• POST (D20) **AFF** 23.89 (SD 7.98)<br>• **Control** group (=) No intervention [mean (SD)]<br>• PRE (D0) **SOM** 29.79 (SD 9.16)<br>• POST (D20) **SOM** 28.95 (SD 9.46)<br>• PRE (D0) **COG** 31.55 (SD 9.04)<br>• POST (D20) **COG** 31.76 (SD 9.23)<br>• PRE (D0) **BEH** 28.69 (SD 9.34)<br>• POST (D20) **BEH** 27.95 (SD 9.68)<br>• PRE (D0) **AFF** 28.93 (SD 9.27)<br>• POST (D20) **AFF** 27.69 (SD 8.67)<br>⇨ **Significant** decrease of score for the somatic, cognitive, behavioral and affective FFAI components within the experimental (=) music intervention group ((p<0.001) for the somatic component; (p<0.01) for the cognitive component; (p<0.01) for the behavioral component; (p<0.02) for the affective component<br>⇨ **Significant** decrease of score for the somatic, cognitive, behavioral and affective FFAI components between the experimental (=) music intervention and control group ((p<0.01) for the somatic component; (p<0.01) for the cognitive component; (p<0.02) for the behavioral component; (p<0.05) for the affective component<br>⇨ **No** significant decrease of score for the somatic, cognitive, behavioral and affective FFAI components within the control group ((p>0.05) for the somatic component; (p>0.05) for the cognitive component; (p>0.05) for the behavioral component; (p>0.05) for the affective component

Title / Author / Year	Questionnaires	Results
**Han** et al. (2011)<br><br>A Controlled Naturalistic Study on a Weekly Music Therapy and Activity Program on Disruptive and Depressive Behaviours in Dementia	**RMBPC**<br>Revised Memory and Behavioral Problems Checklist<br><br>**AES**<br>Apparent Emotion Scale:<br>• Measures 6 types of affect: Pleasure, Anger, Anxiety, Depression, Interest/ Motivation and Contentment	**RMBPC**-Score (Depression sub-scale):<br>• **Experimental** (=) Music & Activity group [mean (SD)]<br>• PRE (Total) (D0) 75.3 (SD 47.6)<br>• PRE (**Depression**) (D0) **20.5** (SD **23.5**)<br>• POST (Total) (W8) 54.5 (SD 40.1)<br>• POST (**Depression**) (W8) **11.7** (SD **15.9**)<br>• **Control** group (=) Usual care [mean (SD)]<br>• PRE (Total) (D0) 62.3 (SD 60.2)<br>• PRE (**Depression**) (D0) **13.1** (SD **21.0**)<br>• POST (Total) (W8) 78.6 (SD 75.7)<br>• POST (**Depression**) (W8) **24.6** (SD **34.7**)<br>⇨ **Significantly** decreased RMBPC total-score within the experimental (=) music therapy & activity group ((p=0.006) (p<0.01))<br>⇨ **Significant** decrease in the depression sub-scale of the RMBPC score at week 8 within the experimental (=) music group ((p=0.019) (p<0.05))<br>⇨ **No** significant decrease in the depression sub-scale of the RMBPC score at week 8 within the control group (p>0.05)<br><br>**AES**-Score (higher is better):<br>• **Experimental** (=) Music therapy & Activity [mean (SD)]<br>• PRE (D0) 18.2 (SD 6.4)<br>• POST (W8) 19.0 (SD 4.8)<br>• **Control** group (=) Usual care (no intervention) [mean (SD)]<br>• PRE (D0) 17.1 (SD 4.3)<br>• POST (W8) 16.6 (SD 5.1)<br>⇨ **No** significant increase in the AES score within the experimental (=) music group (p>0.05) (although a **positive** (nonsignificant) **trend** in favor for the experimental (=) music group score compared to the control group was described)<br>⇨ **No** significant increase in the AES score within the control group (p>0.05)

Title / Author / Year	Questionnaires	Results
**Hanser** and **Thompson** (1994)<br><br>Effects of a music therapy strategy on depressed older adults	**GDS-30**<br>Geriatric Depression Scale (30 items version)<br><br>**BSI-GSI**<br>Brief Symptom Inventory General Severity Index<br><br>**POMS (Bipolar Version)**<br>Profile of Mood States<br>• **Elated-Depressed**<br>• Composed-Anxious<br>• Agreeable-Hostile<br><br>**RSI (=) SEI**<br>Self-Esteem Inventory	**GDS(-30)**-Score:<br>• **Experimental-1** (=) Home-based [mean (SD)]<br>• PRE (D0) 17.30 (SD 5.85)<br>• POST (W8) 07.70 (SD 3.66)<br>• **Experimental-2** (=) Self-administered [mean (SD)]<br>• PRE (D0) 17.60 (SD 7.89)<br>• POST (W8) 12.30 (SD 8.65)<br>• **Control** group (=) Waiting list [mean (SD)]<br>• PRE (D0) 15.30 (SD 5.85)<br>• POST (W8) 16.20 (SD 6.13)<br>⇨ **Significant** decrease in the depression score within the home-based (=) **1st** experimental group ($p<0.05$)<br>⇨ **Significant** decrease in the depression score within the self-administered (=) **2nd** experimental group ($p<0.05$)<br>⇨ **No** significant decrease in the depression score within the control (=) waiting-list group ($p>0.05$)<br><br>**BSI-GSI**-Score:<br>• **Experimental-1** (=) Home-based [mean (SD)]<br>• PRE (D0) 0.85 (SD 0.35)<br>• POST (W8) 0.40 (SD 0.23)<br>• **Experimental-2** (=) Self-administered [mean (SD)]<br>• PRE (D0) 1.18 (SD 0.66)<br>• POST (W8) 0.55 (SD 0.40)<br>• **Control** group (=) Waiting list [mean (SD)]<br>• PRE (D0) 0.71 (SD 0.31)<br>• POST (W8) 0.81 (SD 0.73)<br>⇨ **Significant** decrease in the BSI-GSI score within the home-based (=) **1st** experimental group ($p<0.05$)<br>⇨ **Significant** decrease in the BSI-GSI score within the self-administered (=) **2nd** experimental group ($p<0.05$)<br>⇨ **No** significant decrease in the BSI-GSI score within the control (=) waiting-list group ($p>0.05$)

Title / Author / Year	Questionnaires	Results
**Hanser** and **Thompson** (1994)<br><br>***… continued from above:***<br><br>Effects of a music therapy strategy on depressed older adults	**POMS (Bipolar Version)**<br>Profile of Mood States<br>• **Elated-Depressed**<br>• Composed-Anxious<br>• Agreeable-Hostile	**POMS**-Score (**Elated-Depressed** sub-scale; higher = better):<br>• **Experimental-1** (=) Home-based [mean (SD)]<br>• PRE (D0) 31.67 (SD 06.44)<br>• POST (W8) 47.22 (SD 12.69)<br>• **Experimental-2** (=) Self-administered [mean (SD)]<br>• PRE (D0) 34.78 (SD 08.23)<br>• POST (W8) 44.33 (SD 13.20)<br>• **Control** group (=) Waiting list [mean (SD)]<br>• PRE (D0) 42.20 (SD 08.32)<br>• POST (W8) 37.00 (SD 06.07)<br>⇨ **Significant** increase in the Elated-Depressed [POMS] sub-scale score for the home-based (=) **1st** experimental group ($p<0.05$) (<u>all</u> other POMS (sub-)scores for the 1st group group did also increase significantly ($p<0.05$))<br>⇨ **Significant** increase in the Elated-Depressed score for the self-administered (=) **2nd** experimental group ($p<0.05$) (<u>all</u> other POMS (Sub)-Scores for the 2nd group did also increase significantly ($p<0.05$))<br>⇨ **No** significant increase in the Elated-Depressed score for the waiting list (=) control group ($p>0.05$))
	**RSI (=) SEI**<br>Self-Esteem Inventory	**RSI (=) SEI**-Score:<br>• **Experimental-1** (=) Home-based [mean (SD)]<br>• PRE (D0) 22.00 (SD 7.42)<br>• POST (W8) 17.90 (SD 6.77)<br>• **Experimental-2** (=) Self-administered [mean (SD)]<br>• PRE (D0) 22.10 (SD 5.99)<br>• POST (W8) 19.10 (SD 6.30)<br>• **Control** group (=) Waiting list [mean (SD)]<br>• PRE (D0) 23.10 (SD 5.51)<br>• POST (W8) 22.70 (SD 4.19)<br>⇨ **Significant** decrease of the RSI score for the **Home-based** (=) **1st** experimental group ($p<0.05$)<br>⇨ **Significant** decrease of the RSI score for the **Self-administered** (=) **2nd** experimental ($p<0.05$)<br>⇨ **No** significant decrease in the Elated-Depressed score for the w**aiting list** (=) control group ($p>0.05$)

Title / Author / Year	Questionnaires	Results
**Harmat** et al. (2008)<br><br>Music improves sleep quality in students	**BDI**<br>Beck's Depression Inventory<br><br>**PSQI**<br>Pittsburgh Sleep Quality Index<br><br>**ESS**<br>Epworth Sleepiness Scale<br>⇨ <u>Initial</u> selection only!	**BDI**-Score:<br>• **Experimental-1** (=) Music (listening) [mean (SD)]<br>• PRE (D0) 5.40 (SD 3.767)<br>• POST (W03) 2.66 (SD n/a)<br>• **Experimental-2** (=) Audiobook (listening) [mean (SD)]<br>• PRE (D0) 5.70 (SD 3.564)<br>• POST (W03) 5.13 (SD n/a)<br>• **Control** group [mean (SD)]<br>• PRE (D0) n/a (SD n/a)<br>• POST (W03) n/a (SD n/a)<br>⇨ **Significant** decrease in the score for depression within the **1st** experimental (=) **music** group (p<0.05)<br>⇨ **No** significant decrease in the score for depression within the **2nd** experimental (=) **audiobook** group (p>0.05)<br>⇨ **No** significant decrease in the score for depression within the control group (p>0.05)<br><br>**PSQI**-Score (lower scores (=) healthier sleep quality):<br>• **Experimental-1** (=) Music (listening) [mean (SD)]<br>• PRE (D0) 6.83 (SD 2.093)<br>• POST (W03) 3.27 (SD 1.800)<br>• **Experimental-2** (=) Audiobook (listening) [mean (SD)]<br>• PRE (D0) 6.27 (SD 1.721)<br>• POST (W03) 5.17 (SD 2.214)<br>• **Control** group [mean (SD)]<br>• PRE (D0) n/a (SD n/a)<br>• POST (W03) n/a (SD n/a)<br>⇨ **Significant** decrease in the score for sleep quality within the **1st** experimental (=) **music** group (p<0.05)<br>⇨ **No** significant decrease in the score for sleep quality within the **2nd** experimental (=) **audiobook** group (p>0.05)<br>⇨ **No** significant decrease in the score for sleep quality in the control group (p>0.05)

Title / Author / Year	Questionnaires	Results
**Hendricks** et al. (1999)<br><br>Using Music Techniques to Treat Adolescent Depression	**BDI**<br>Beck's Depression Inventory	**BDI**-Score:<br>• **Experimental** (=) Music group [mean (SD)]<br>• PRE (D0) 39.00 (SD n/a)<br>• POST (D20) 01.34 (SD n/a)<br>• **Control** group [mean (SD)]<br>• PRE (D0) 32.30 (SD n/a)<br>• POST (D20) 17.00 (SD n/a)<br>⇨ **Significant** decrease in the score for depression within the experimental (=) music group ((p=0.0195) (p<0.05))<br>⇨ **Significant** decrease in the score for depression within the control group (p<0.05)
**Hsu** and **Lai** (2004)<br><br>Effects of Music on Major Depression in Psychiatric Inpatients	**SDS**<br>(Zung's) Self-Rating Depression Scale<br>• Sub-Scores:<br>• Pervasive-Affective disturbances<br>• Physiological disturbances<br>• Psychological disturbances<br>• Psychomotor disturbances	**SDS**-Score:<br>• **Experimental** (=) Music group [mean (SD)]<br>• PRE (D0) 81.34 (SD 6.39)<br>• POST (W2) 51.39 (SD 6.21)<br>• **Control** group (=) Bed rest [mean (SD)]<br>• PRE (D0) 80.60 (SD 5.34)<br>• POST (W2) 62.17 (SD 7.07)<br>⇨ **Significant** decrease in the SDS score for depression within the experimental (=) music group (p<0.001)<br>⇨ **Significant** better **global** depressive SDS scores within the experimental (=) music group compared to the control group at each time point (p<0.05)<br>⇨ **Significant** decrease in the SDS scores for depression on all of the **four sub**-scores over two weeks within the experimental (=) music group<br>• Pervasive-Affective disturbances<br>• Physiological disturbances (p<0.05)<br>• Psychological disturbances (p<0.05)<br>• Psychomotor disturbances (p<0.05)<br>⇨ **Significant** decrease in the SDS scores for depression within the experimental (=) music group compared to the control group at each time point (p<0.05)<br>⇨ **No** significant decrease in the score on depression within the control (=) resting group (p>0.05)

Title / Author / Year	Questionnaires	Results
**Kim** et al.<br>(2006)<br><br>The Effect of Music Therapy on Anxiety and Depression in Patients Undergoing Hemodialysis	**SDS**<br>(Zung's) Self-Rating Depression Scale	**SDS**-Score:<br>• **Experimental** (=) Music group ([mean (SD)]<br>• PRE (D0) 46.11 (SD 8.67)<br>• POST (W2) 42.17 (SD 8.44)<br>• **Control** group (=) Bed rest [mean (SD)]<br>• PRE (D0) 46.44 (SD 8.29)<br>• POST (W2) 47.44 (SD 9.12)<br>⇨ **Significant** decrease in the SDS score for depression within the experimental (=) music group ($p<0.01$)<br>⇨ **No** significant decrease in the SDS score for depression within the control group ($p>0.05$)

Title / Author / Year	Questionnaires	Results
**Koelsch** et al. (2010)<br><br>Music in the treatment of affective disorders: an exploratory investigation of a new method for music-therapeutic research	**POMS (Brief Version)**<br>• Short 35-item version<br>• German translation<br><br>(**Basic-)Emotion-Score**: (during the experiment)<br>• Likert scales from **0.00** ("not at all") up to **8.00** ("very strongly")<br><br>**SAMs**<br>Modified (9-point) Self-Assessment Manikins<br>⇨ Valence & Arousal<br><br>**TAS-26**<br>Toronto Alexithymia Scale<br>⇨ Pretest before the experiment only<br><br>**Affective disorders**:<br>• Depression<br>• Fatigue<br>• Vigor<br>• Irritability	**POMS**-Score (Depression sub-scale):<br>• **Experimental** (=) Music group [mean (SD)]<br>• PRE (n/a) 6.57 (SD 08.01)<br>• POST (n/a) 3.51 (SD 05.91)<br>• **Control** group (=) Tapping to a beat [mean (SD)]<br>• PRE (n/a) 8.25 (SD 10.37)<br>• POST (n/a) 9.30 (SD 9.68)<br>⇨ **Significant** decrease in the depression [POMS] sub-scale score within the experimental (=) music group ($p<0.001$)<br>⇨ **No** significant decrease in the score on depression in the control (=) "tapping to a beat" group (($p=0.33$) ($p>0.05$))<br>⇨ **Significant** group differences tested separately for pre and post measures ($p<0.001$)<br><br>**(Basic-)Emotion**-Score (Happiness sub-scale):<br>• **Experimental** (=) Music group [mean (SD)]<br>• Happiness 4.69 (SD 1.64)<br>• **Control** group (=) Tapping to a beat [mean (SD)]<br>• Happiness 2.09 (SD 1.11)<br>⇨ **Significant** group differences tested separately for pre and post measures ($p<0.0001$)<br>⇨ **Significant** better score in the experimental (=) music compared to the control (=) "tapping to a beat" group<br><br>**SAMs**-Score (Valence & Arousal sub-scales):<br>• **Experimental** (=) Music group [mean (SD)]<br>• Valence 1.90 (SD 0.95)<br>• Arousal 4.50 (SD 1.15)<br>• **Control** group (=) Tapping to a beat [mean (SD)]<br>• Valence 0.10 (SD 0.92)<br>• Arousal 3.99 (SD 1.23)<br>⇨ **Significant** difference between the experimental (=) music compared to the control (=) "tapping to a beat" group for the valence emotion rating ($p<0.0001$)<br>⇨ **No** significant difference between the experimental (=) music compared to the control (=) "tapping to a beat" group for the arousal emotion ratings ($p>0.05$)

Title / Author / Year	Questionnaires	Results
**Lu** et al. (2013)<br><br>Effects of group music intervention on psychiatric symptoms and depression in patient with schizophrenia	**CDSS**<br>Calgary Depression (*Rating*) Scale for Schizophrenia<br><br>**PANSS**<br>Positive and Negative Syndrome Scale<br>⇨ Measuring psychotic symptom severity of patients with schizophrenia	**CDSS**-Score:<br>• **Experimental** (=) Music intervention group [mean (SD)]<br>• PRE (W0) 4.23 (SD 4.78)<br>• POST (W5) 0.89 (SD 1.62)<br>• **Control** group (=) Usual care [mean (SD)]<br>• PRE (W0) 3.38 (SD 4.65)<br>• POST (W5) 3.33 (SD 4.25)<br>⇨ **Significant** decrease in the score for depression within the experimental (=) music group ((p=0.004) (p<0.005))<br>⇨ **No** significant decrease in the depression score within the control group (p>0.05)<br><br>**PANSS(-total)**-Score:<br>• **Experimental** (=) Music intervention group [mean (SD)]<br>• PRE (W0) 73.89 (SD 19.14)<br>• POST (W5) 65.77 (SD 18.19)<br>• **Control** group (=) Usual care [mean (SD)]<br>• PRE (W0) 71.30 (SD 22.56)<br>• POST (W5) 80.80 (SD 22.73)<br>⇨ **Significant** decrease in the [PANSS] score within the experimental (=) music group (p<0.001)<br>⇨ **No** significant decrease in the [PANSS] score within the control group (p>0.05)

Title / Author / Year	Questionnaires	Results
**Schwantes** and **McKinney** (2010)<br><br>Music therapy with Mexican migrant farmworkers: A pilot study	**CES-D**<br>Center for Epidemiological Studies Depression Scale (Spanish version)<br><br>**BSI-18**<br>Brief Symptom Inventory-18	**CES-D**-Score:<br>• **Experimental** (=) Music group [mean (SD)]<br>• PRE (W0) 21.60 (SD 3.22)<br>• POST (W4) 15.60 (SD 2.66)<br>• **Control** group [mean (SD)]<br>• *No control used*<br>⇨ **Significant** decrease in the score for depression within the experimental (=) music group ((p=0.013) (p<0.05))<br><br>**BSI-18**-Score:<br>• **Experimental** (=) Music group [mean (SD)]<br>• PRE (W0) 12.20 (SD 4.32)<br>• POST (W4) 07.00 (SD 2.19)<br>• **Control** group [mean (SD)]<br>• *No control used*<br>⇨ **No** significant decrease in the BSI(-18) score within the experimental (=) music group ((p=0.379) (p>0.05))
**Silverman** (2011)<br><br>Effects of Music Therapy on Change and Depression on Clients in Detoxification	**BDI**<br>Beck's Depression Inventory<br><br>**Seven point Likert-type Scale** (for Treatment perception(s) rating)<br><br>**Questionnaire**<br>(about perception(s) of enjoyment and comfort during each session)<br><br>Info: A randomized, two group **post-test only design** in addition to a follow-up after 1 month, was used for this study.	**BDI**-Score:<br>• **Experimental** (=) Music group [mean (SD)]<br>• Posttest 18.79 (SD 9.14)<br>• **Control** group (=) Verbal therapy [mean (SD)]<br>• Posttest 20.28 (SD 9.53)<br>⇨ **No** significant decrease in the score for depression within the experimental (=) music group (p>0.05)<br>⇨ **No** significant difference in BDI-Scores between the experimental (=) music and the the control (=) verbal therapy group (p>0.05)<br>⇨ **No** significant decrease in the score for depression within the control (=) verbal therapy group (p>0.05)

Title / Author / Year	Questionnaires	Results
**Verrusio** et al. (2014)<br><br>Exercise training and music therapy in elderly with depressive syndrome: a pilot study.	**GDS-15**<br>Geriatric Depression Scale (15 items version)<br><br>**HAM-A** (=) **HAS**<br>Hamilton **Anxiety** Rating Scale<br><br>**CIRS**<br>Cumulative Illness Rating Score<br>⇨ Initial selection only!<br><br>**CInd**<br>Comorbidity Index<br>⇨ Initial selection only!	**GDS(-15)**-Score:<br>• **Experimental** (=) Music group [mean (SD)]<br>• PRE (W0) 8.5 (SD 2.2)<br>• POST (W24) 5.5 (SD 1.0)<br>• **Control** (=) Pharmacotherapy group [mean (SD)]<br>• PRE (W0) 8.4 (SD 1.8)<br>• POST (W24) 8.0 (SD 2.5)<br>⇨ **Significant** decrease in the score for depression within the experimental (=) music group (p<0.01)<br>⇨ **No** significant decrease in the score for depression within the control (=) pharmacotherapy group (p>0.05)<br><br>**HAM-A**-/ (=) **HAS**-Score:<br>• **Experimental** (=) Music group [mean (SD)]<br>• PRE (W0) 22.2 (SD 5.07)<br>• POST (W24) 16.5 (SD 2.70)<br>• **Control** (=) Pharmacotherapy group [mean (SD)]<br>• PRE (W0) 24.2 (SD 5.30)<br>• POST (W24) 22.0 (SD 4.70)<br>⇨ **Significant** decrease in the score for anxiety within the experimental (=) music group (p<0.001)<br>⇨ **No** significant decrease in the score for anxiety within the control (=) pharmacotherapy group (p>0.05)

Title / Author / Year	Questionnaires	Results
**Wang** et al. (2011)<br><br>Impact of group music therapy on the depression mood of college students	**SDS**<br>(Zung's) Self-Rating Depression Scale<br><br>**SCL-90**<br>Symptom Checklist 90 (Chinese translation)	**SDS**-Score:<br>• **Experimental** (=) Group music-therapy [mean (SD)]<br>• PRE (D0) 45.45 (SD 5.23)<br>• POST (n/a) 40.21 (SD 5.10)<br>• **Control** group (=) No therapy [mean (SD)]<br>• PRE (D0) 46.01 (SD 5.34)<br>• POST (n/a) 46.19 (SD 5.07)<br>⇨ **Significant** decrease in the score on depression within the experimental (=) music intervention group (p<0.001)<br>⇨ **No** significant decrease in the score on depression within the control group (p>0.05)<br><br>**SCL-90**-Score:<br>• **Experimental** (=) Music group [mean (SD)]<br>• PRE (D0) 214.32 (SD 39.24)<br>• POST (n/a) 198.73 (SD 44.11)<br>• **Control** group (=) No therapy [mean (SD)]<br>• PRE (D0) 213.35 (SD 38.93)<br>• POST (n/a) 214.83 (SD 45.39)<br>⇨ **Significant** decrease in the SCL-90 score within the experimental (=) music group (p<0.001)<br>⇨ **No** significant decrease in the SCL-90 score within the control group (p>0.05)<br>⇨ **Significant** difference of SCL-90 scores between the experimental (=) music and the control group (p<0.001)
